# Educational interventions to improve people’s understanding of key concepts in assessing the effects of health interventions: a systematic review

**DOI:** 10.1186/s13643-018-0719-4

**Published:** 2018-05-02

**Authors:** Leila Cusack, Chris B. Del Mar, Iain Chalmers, Elizabeth Gibson, Tammy C. Hoffmann

**Affiliations:** 1 Centre for Research in Evidence-Based Practice (CREBP), Faculty of Health Sciences and Medicine, Bond University, 14 University Drive, Robina, QLD 4229 Australia; 2 James Lind Initiative, Oxford, UK

**Keywords:** Consumer, Education, Health information, Health literacy, Critical health literacy, Critical appraisal, Critical thinking

## Abstract

**Background:**

Health information is readily accessible but is of variable quality. General knowledge about how to assess whether claims about health interventions are trustworthy is not common, so people’s health decisions can be ill-informed, unnecessarily costly and even unsafe. This review aims to identify and evaluate studies of educational interventions designed to improve people’s understanding of key concepts for evaluating claims about the effects of health interventions.

**Methods/Design:**

We searched multiple electronic databases and sources of grey literature. Inclusion criteria included all study types that included a comparison, any participants (except health professionals or health professional students) and educational interventions aimed at improving people’s understanding of one or more of the key concepts considered necessary for assessing health intervention claims. Knowledge and/or understanding of concepts or skills relevant to evaluating health information were our primary outcome measures. Secondary outcomes included behaviour, confidence, attitude and satisfaction with the educational interventions. Two authors independently screened search results, assessed study eligibility and risk of bias and extracted data. Results were summarised using descriptive synthesis.

**Results:**

Among 24 eligible studies, 14 were randomised trials and 10 used other study designs. There was heterogeneity across study participants, settings and educational intervention type, content and delivery. The risk of bias was high in at least one domain for all randomised studies. Most studies measured outcomes immediately after the educational intervention, with few measuring later. In most of the comparisons, measures of knowledge and skills were better among those who had received educational interventions than among controls, and some of these differences were statistically significant. The effects on secondary outcomes were inconsistent.

**Conclusions:**

Educational interventions to improve people’s understanding of key concepts for evaluating health intervention claims can improve people’s knowledge and skills, at least in the short term. Effects on confidence, attitude and behaviour are uncertain. Many of the studies were at moderate or greater risk of bias. Improvements in study quality, consistency of outcome measures and measures of longer-term effects are needed to improve confidence in estimates of the effects of educational interventions to improve people’s understanding of key concepts for evaluating health intervention claims.

**Systematic review registration:**

PROSPERO CRD42016033103

**Electronic supplementary material:**

The online version of this article (10.1186/s13643-018-0719-4) contains supplementary material, which is available to authorized users.

## Background

Health information and misinformation are readily accessible to the general public, particularly through mass media and the Internet [1–7]. Due to the ease with which large amounts of health information can now be accessed, people are playing a more active and autonomous role in their health [1, 8]. The health information that people access can affect their health decisions and behaviours—for example, from how they maintain their health and cope with a chronic condition to decisions made about how to treat an illness or whether to consult a health professional [9, 10].

As well as websites and traditional information sources such as magazines, radio and television, health information is also available on social media such as Facebook [11], YouTube health channels and Twitter [7, 12]. Regardless of the type of media, health information remains essentially unregulated, and problems and concerns about its quality have been noted [7, 13–15]. Consequently, people are exposed to and have to navigate vast amounts of health information, often on their own, typically with little knowledge about how to evaluate it or the need to do so [16].

Health interventions (an umbrella term for anything that can potentially change a health outcome, such as medical treatments, food and drinks, beauty products, exercise or therapy and behaviour changes) are one of the most commonly researched health topics [9, 10], yet the quality of health information is variable [5]. Some health interventions are promoted using phrases such as ‘evidence-based’ and ‘clinically proven’. Phrases such as these are intended to convince people of a health intervention’s effectiveness, so when the claims are unwarranted, they can be misleading. Some of the other complexities that can influence people’s decision-making about health interventions are that some people tend to rely on anecdotes rather than information derived from research [17, 18]. These can overrate the trustworthiness of the health information they find, and most overestimate the benefits and underestimate the harms of health interventions [19]. Belief in false or unsubstantiated claims about health interventions may result in people receiving inappropriate health interventions that are at best ineffective, at worst harmful, as well as wasteful of healthcare resources. Conversely, not believing claims that are based on reliable research evidence about beneficial health interventions can also cause harm, for example, through inappropriate treatments or delays in seeking appropriate health care [20–24] or choosing ineffective treatments over effective ones [25].

Health information may be misleading, misinterpreted or leave people confused [13, 26]. People require basic skills to assess the quality of both general health information and information about health interventions and their effects. Without education about key concepts relevant to evaluating the effects of health interventions and how to interpret research results, people are, irrespective of their level of education, vulnerable to believing untrustworthy treatment claims and may make health decisions based upon information that is inaccurate, incomplete or even harmful [21, 25]. Because of its nature and extensive reach, health information on the Internet is impossible to regulate. However, providing people with knowledge about key concepts in evaluating information about health interventions may help them evaluate the trustworthiness of health intervention claims and to make informed decisions. The general public is typically not trained to evaluate the accuracy and completeness of information about health interventions [16].

Most of the existing research on helping people to understand health information has focused on the traditional skills associated with health literacy, such as reading, numeracy and understanding speech. Limitations in these skills can impact upon people’s ability to navigate the health system and are associated with poorer outcomes and decreased uptake of health services [27]. Previous systematic reviews have examined the effectiveness of related educational interventions such as teaching online health literacy to the general public [27], critical appraisal skills to health professionals [28] and the effects of educational interventions on critical appraisal abilities in school students [29]. As far as we are aware, however, there is no systematic review of studies of educational interventions designed to improve critical appraisal abilities in the general public. The aim of the current systematic review is to assess the effectiveness of educational interventions designed to improve people’s understanding of key concepts (described below) when evaluating claims about the effects of health interventions.

## Methods

The methodology of this review has been described in detail in a previously published protocol through PROSPERO (CRD42016033103) [30]. Key information is presented here, along with any clarifications and modifications of the methods described in the published protocol.

### Information sources

We searched for reports of relevant research using the Cochrane Central Register of Controlled Trials (CENTRAL, The Cochrane Library, Issue 10 of 12, October 2015), MEDLINE (1946 to November 2015), EMBASE (1966 to November 2015), CINAHL (1982 to November 2015), ERIC (1990 to November 2015) and the Critical thinking and Appraisal Resource Library (CARL) database [31] (Castle et al. 2017) (from its creation in 2016 until January 2018). The search strategies were designed specifically for each database and informed by the strategy used in the review of critical appraisal educational interventions for school students [29]. Forward and backward searches of eligible reports were performed. The full search strategy and an example search in MEDLINE are detailed in the protocol [30]. Searches for grey literature included contacting researchers in the field.

### Eligibility criteria

#### Study types and participants

Studies with the following designs were eligible for inclusion: randomised trials, non-randomised trials with concurrent controls, controlled before and after studies, controlled studies with only post-test measures and interrupted time series studies. All types of study participants (learners) were eligible except university students undertaking a health professional degree and health professionals. Health students and/or health professionals were excluded from the review, as an educational intervention provided to them on this topic is most likely designed to assist them to perform decision-making with their patients, rather than decision-making regarding their own health. The following clarifications were specified once eligibility criteria had been applied: (1) if a mixed population was identified (e.g. a combination of eligible and ineligible participants), the study was only eligible if the results of the eligible populations were reported or available separately, and (2) the definition of a ‘health professional’ was clarified as any profession regulated by the Australian Health Practitioner Regulation Agency (AHPRA) [32] and likewise, a ‘tertiary health student’ was specified as any student who was enrolled in courses as part of a health degree which would result in graduation as one of the AHPRA-listed health professions as these students are likely to have received education in evidence-based practice (EBP). Studies in which participants were students undertaking a university course about health but were not enrolled in a degree course where they would graduate as a health professional (as defined above) were considered eligible.

#### Interventions

All educational interventions were eligible if they aimed to help the participants (learners) understand one or more of the key concepts considered relevant to evaluating the effects of health interventions and/or the interpretation of research results, as long as the examples/scenarios used were within the context of health information or claims, health conditions, the human body and/or conventional, complementary or alternative healthcare treatments. The main indicators of relevance were general topics such as evidence-based skills, the evidence-based process, scientific reasoning, critical health literacy skills, basic research concepts, randomised trial concepts, assessing claims about health treatments and specific topics such as randomisation, blinding, causation, placebo, statistical reasoning and understanding risk. There was no restriction on other characteristics of the educational interventions. During the eligibility assessment process, we clarified that studies designed to improve informed consent about participating in a specific clinical trial would not be eligible.

#### Outcomes

Our primary outcomes were any measure of knowledge or understanding of concepts or skills relevant to evaluating the effects of, or claims about, health interventions, as well as any measures in which some questions assessed knowledge and some assessed skills. At the data extraction stage, it was clarified that as well as single measures (such as a measure of knowledge), the primary outcome could be any measure that assessed a combination of these outcomes (e.g. a combined measure of knowledge and skills). In our protocol, we had stated that skills would be one of our secondary outcomes. Secondary outcomes were any measures of application of the knowledge taught, through demonstrations of relevant behaviours and attitudes, as well as confidence, and satisfaction with the educational intervention. Our outcomes reflect the first three of the four levels used in the Kirkpatrick evaluation model for evaluating training effectiveness [33]: (1) reaction, (2) learning and (3) behaviour.

### Study selection

Two authors (LC and EG) independently screened the titles and abstracts to identify potentially eligible reports. Initially, each reviewer independently screened a sample of approximately 50 citations to ensure adequate inter-rater agreement. Both researchers read the full text of potentially eligible reports to assess whether they met our eligibility criteria. Unresolved study eligibility decisions were clarified through consultation with a third author (TH).

### Data collection

Data were extracted and entered into a customised data collection spreadsheet by two authors (LC and EG) independently. Each of the two authors initially independently extracted data for five reports and then compared and discussed this to ensure inter-rater agreement. Small modifications to the layout of the data extraction spreadsheet were made and prompts added before extraction continued. Any discrepancies were discussed and resolved, with input from a third author (TH) when consensus could not be reached. Data items extracted are detailed in the review protocol [30] and shown in Additional files 1, 2 and 3: Tables S1–S3.

### Risk of bias in included studies

All studies were quality assessed, using either the Cochrane Collaboration’s tool for assessing the risk of bias in randomised trials [34] or the ROBINS-I tool (a tool to assess the risk of bias in non-randomised studies) [35], as appropriate. Although the protocol indicated that we would be using ACROBAT NRSI, an updated version of the ROBINS-I tool became available after the protocol had been developed.

### Data synthesis

Because of the heterogeneity of learners, study designs, educational intervention designs and type and outcomes across included studies, results were reported descriptively.

## Results

**Fig. 1:**
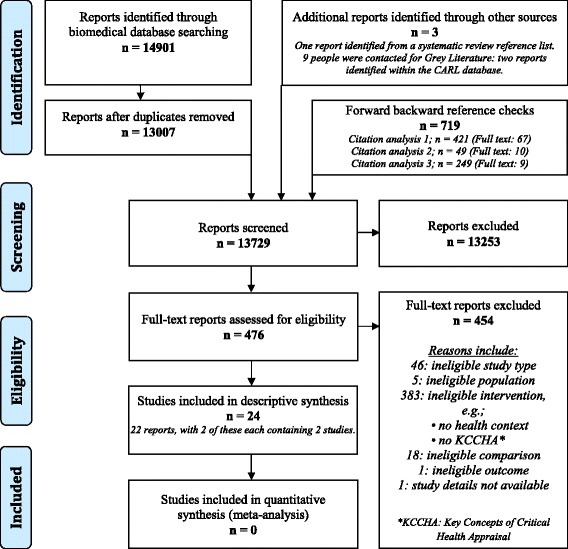
PRISMA flow chart of reports through the review searching and inclusion process

Figure 1 shows the flow of articles during the review process. The search identified 14,901 reports, and 13,007 remained after removing duplicates. After screening titles and abstracts, the full text of 476 reports were examined, and 22 of these were judged to contain a total of 24 eligible studies.

### Study characteristics

Two of the 22 eligible articles [36, 37] each included reports of two randomised trials [37–40]. Of the 24 eligible studies, 14 were randomised studies [37–50], and the remaining 10 were other eligible study types [51–60]. One of the included articles [60] was published in German and translated using Google translate and checked by a German language speaker. Characteristics of the studies are shown in Additional file 1: Table S1. With the exception of the large study by Nsangi et al. [37], which involved 12,639 primary school students, sample sizes ranged from 36 [46] to 1465 [57] participants. Most studies used convenience sampling. Of those that did not, Ndebele [46] used purposive sampling to include participants with demonstrated low understanding of randomisation, double-blinding and placebo; Nsangi 2017a [37] used multi-stage stratified random sampling to include students in grade 5, Nsangi 2017b [38] used purposive sampling to include grade 5 teachers from within the cluster randomisation of classrooms and Rowe [56] used a combination of retrospective and convenience sampling to compare the results from multiple semesters of university courses following the educational intervention with others following the standard course over 5 years. One study included four intervention arms (comparing different educational intervention formats) [45].

The study design of two studies was particularly complex, which has implications for how the studies were categorised in this review and had their risk of bias assessed [44, 57]. Although the Hendricks study [44] reported using random assignment, some tasks and outcomes involved participants who were not randomised. As the outcome measures from this component of the study were not eligible or extracted for this review, this study was categorised as a randomised study. In the Kaelin study [57], although teachers who volunteered to participate were randomly allocated to one of the two conditions (and some analyses were of the randomised groups), a group of non-volunteer teachers was also included and treated as controls. We categorised this study as non-randomised.

#### Settings

Seven studies took place in university settings, with four in the USA [36, 55, 56, 58], two in Germany [54, 60] and one in Canada [53]. Seven studies occurred in formal school settings in classrooms (age range 9–20 years, grades 5–12), with five in the USA [42, 44, 51, 57, 59], one in Germany [52] and one in Uganda [37]. Three studies involved web-based modules and were based in the USA [50], Norway [41] and a multi-country study involving Canada, Norway, Argentina and Italy [47]. Three studies were set in outpatient clinics, with two based in the USA [36, 49] and one in Denmark [45]. Four studies were conducted in community-based settings such as meeting rooms in the USA [43], Malawi [46] and Uganda [38] and in participants’ homes and workplaces in Uganda [48].

#### Participant characteristics

With the exception of one study [46], studies included learners of both genders. Participants’ gender was not given for five studies [42, 51, 56, 58, 60]; nine reported > 60% female learners [39, 41, 45–48, 52, 54, 55], four studies reported 50–60% female learners [44, 50, 53, 57], five studies reported < 50% female learners [37, 40, 43, 59] and one study reported two populations, parents and children (67 and 50% respectively) [49]. Ethnicity was reported in nine studies, with seven of these comprising a majority of Caucasian participants (ranging from 57 to 99%) [39, 40, 43, 49, 52, 56, 59], while Hispanic learners were a majority (54%) in one study [57]. The remaining study did not provide specific details, other than to say that the ethnic inclusion was ‘diverse’ [51]. Three studies were performed in Uganda [37, 38, 48]. Only three studies detailed socioeconomic status [39, 40, 44].

Children and/or adolescents (still in primary or secondary school) were participants in seven studies [37, 42, 44, 51, 52, 57, 59], and young adults (of university age) were participants in five studies [53, 55, 56, 58, 60]. Adults were participants in ten studies [38–41, 43, 45–48, 54]. Two studies reported on mixtures of adults and children [49] and young adults and adults [50].

#### Intervention details

The content and format of educational interventions varied considerably across studies (see Additional file 1: Table S1). Twelve studies provided the educational intervention through existing avenues of education (such as school or university) [37, 42, 44, 51, 53, 55–60]. These educational interventions ranged in duration from a leaflet handed out on one occasion [42] to courses run over a few days to a week [44, 52, 60] and to courses spanning multiple weeks or across a school or university semester [37, 38, 48, 53, 55, 56, 58]. In the school- and university-based studies, a mixed approach to teaching was common [44, 59] and included interactive sessions, small group work, class discussions and didactic components.

Eight studies relied on independent learning, either in the form of reading a leaflet or booklet [39, 40, 45], or by using an interactive web-based program [41, 47, 49, 50] or podcast [48]. Four studies evaluated educational interventions designed to enhance public education for adults outside formal institutional learning; these were held in meeting rooms or university classrooms and ranged from a single session [43, 61] to a week-long course [54], and courses lasting multiple weeks [48]. For educational interventions that were delivered face-to-face, educational intervention providers included high school teachers [37, 44, 51, 57], university lecturers [53, 55, 56] and members of a research team [38, 52, 54, 59].

#### Reported outcomes

Of the 136 outcome measures provided (some of the outcome measures/tools provided multiple results for each outcome), 51 assessed knowledge or knowledge and skills and included one unusual outcome that assessed skills and behaviour combined [54] (see Additional file 2: Table S2), and 85 assessed confidence, perception of (one’s own) skills, attitude, behaviour or satisfaction (see Additional file 3: Table S3).

In two studies [39, 40], the same study design had been used by the same team in two different populations. Three other linked studies [37, 38, 48] used a similar basis for the educational intervention and the same outcome assessment. Apart from these exceptions, there was no consistency among the measurements used to assess outcomes. In some studies, the outcome measurement tools used had some form of validation (see Additional files 2 and 3: Table S2 and Table S3). Some studies used a combination of validated measures (or measures adapted from validated measure) and measures developed specifically for that study; others used only measures developed for that study with no description of validation.

The timing of the measurement of outcomes varied. Of the total 64 outcomes, 41 were measured only after the educational intervention, with 39 immediately or shortly following the educational intervention. Twenty-three measurements included assessments before and after the educational interventions, and in 18 of these, measurements were made immediately or shortly after the educational intervention.

### Risk of bias in included studies

**Fig. 2:**
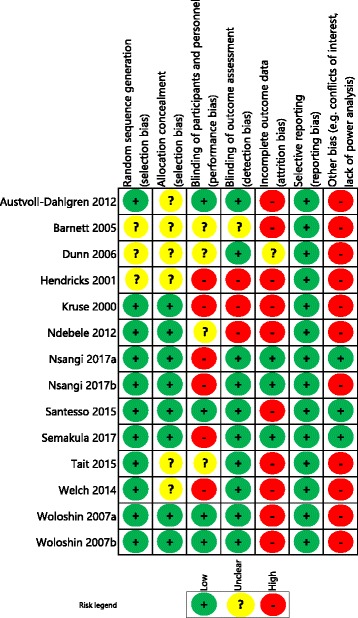
Summary of risk of bias assessment for the 14 studies using randomised controls

**Fig. 3:**
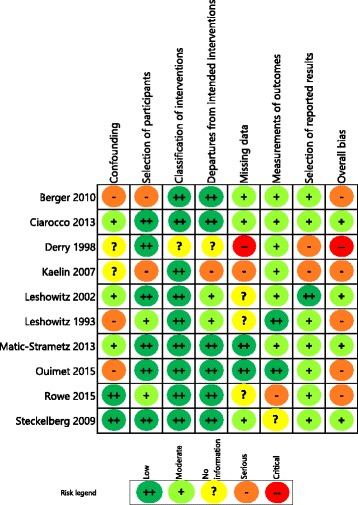
Summary of risk of bias assessment in 10 studies using non-random allocation of controls

Although there was variation in the risk of bias of the included studies, the majority (22 studies) contained at least one domain classified as at high (or serious) risk of bias or no information. The risk of bias summary for each study is presented separately for randomised trials (Fig. 2) and other study types (Fig. 3).

#### Randomised trials

All the studies using randomised controls were considered to be at high risk of at least one area of bias other than allocation bias (Fig. 2). Attrition bias was a common area of high risk, as only three randomised studies reported an intention-to-treat analysis [37, 45, 48]. Performance bias was another area of high risk, with six studies being classified at high risk [37, 38, 44, 45, 48, 50] and four as at low risk of bias [39–41, 47], with the remaining four studies as at unclear risk. Blinding of participants and assessors was often not possible given the nature of the educational interventions and self-reported outcome measures.

#### Other study types

Of the ten studies that had used non-randomised designs (Fig. 3), four were controlled before and after studies [53, 56, 58, 60], another four were controlled studies with only post-test measures [52, 54, 55, 59] and two used concurrent controls [51, 57]. Four were considered to be at moderate overall risk [52, 55, 58, 60] and six to be at serious or critical risk of bias overall [51, 53, 54, 56, 57, 59]. The reasons for the high risk of bias varied across the studies, ranging from missing participant numbers and/or missing data [51], lack of pre-test [59], differences between incentives offered [56] or differences between the groups, notably the inclusion of learners with a pre-existing interest in the topic [54].

### Effects of educational interventions

**Fig. 4:**
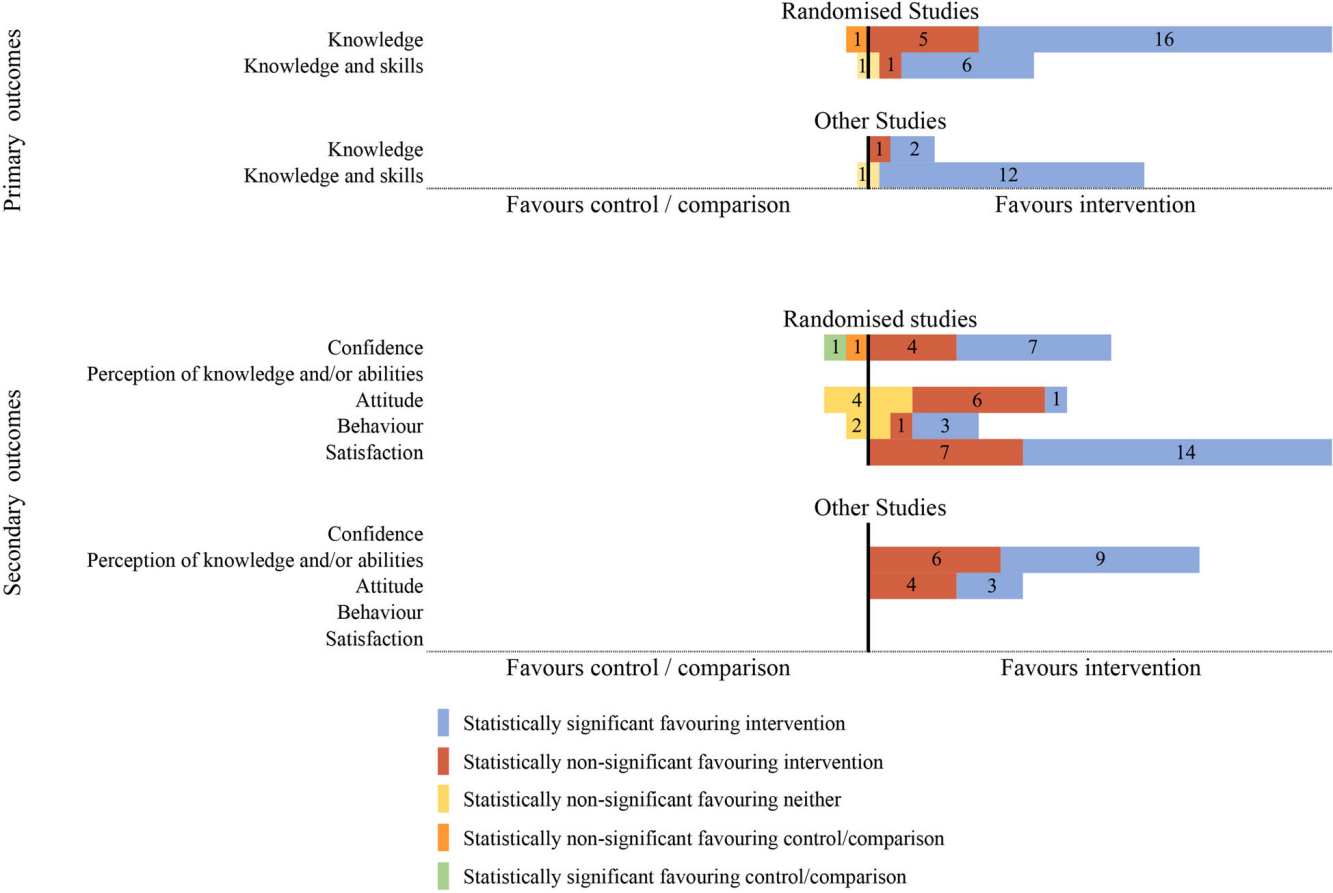
Summary graph of between-group measurements across all studies

Additional file 2 Table S2 shows the studies that assessed the review’s primary outcomes and their results. Additional file 3: Table S3 details these for secondary outcomes. Meta-analysis was precluded due to the heterogeneity among studies. The majority of the included studies provided between-group comparisons of an intervention and control, enabling us to summarise our findings. A summary of the between-group results is shown in Fig. 4, with results presented separately for primary and secondary outcomes, and for randomised trials and other study designs. Results were excluded from this summary where there was either no between-group comparison (e.g. only within-group pre-post testing), a comparison between different formats of the educational intervention (such as booklet vs leaflet) or where the measure was only completed for the educational intervention group and lacked a control group for comparison (such as measures of satisfaction with the educational intervention). Although the studies themselves met our criteria, some of the (often multiple) outcome measures did not.

### Primary outcomes

#### Knowledge

Of the 24 measures of knowledge (from seven randomised studies), statistically significantly better results for 16 measures were found in educational intervention group participants compared to those in control groups. These included measures of understanding double blinding, placebo, implications and composite scores of these topics [46]; understanding quality of evidence and its components [47]; understanding basic research concepts (pre-post and post-post) and knowledge of clinical trial participation [49]; understanding placebos [43]; knowledge about causality [44]; and evidence-based practice [50]. Five measures of knowledge from three randomised studies [46, 47, 49] favoured the educational intervention group but were not statistically significant, while another measure of knowledge favoured the control group but was also not statistically significant. One non-randomised study provided two statistically significant outcomes and one statistically non-significant outcome, all favouring the intervention [57].

#### Knowledge and skills

Eight randomised studies used nine measures of knowledge and skills. Across six measures of knowledge and skills used in six of these eight studies, there was statistically significantly better performance in educational intervention groups than among controls. These included participants’ ability to appraise claims about treatments [37, 38, 48] and ability to apply the principle of causality [44]. The other two randomised studies assessed the ability of learners to interpret medical statistics [39, 40]. The same educational intervention and measures were used for both studies, the only difference being the socioeconomic status (SES) of the populations. Both studies found statistically significantly better ability in the educational intervention group participants than in control group participants (6 and 7 points difference for the high and low SES respectively on adjusted analysis). One randomised study assessed two measures of knowledge and skills; both results were statistically non-significant, with one finding better results after the educational intervention than in the control group, and the other favouring neither intervention nor control [41]. The remaining study compared different formats and has not been included in the summary figure (Fig. 4).

Eight studies using designs other than randomised trials assessed knowledge and skills, but each study used a different measure (15 measures in total) and each evaluated different knowledge and skills. Results for 12 of the measures were statistically significantly better in educational intervention groups than in comparison groups. These included measures of critical thinking and scientific reasoning [59], methodological reasoning and evaluating study quality [58], critical appraisal skills [53, 60], critical thinking [56] and critical health literacy competence [52].

#### Skills and behaviour (combination of primary and secondary outcomes)

One non-randomised two group comparison assessed the long-term implementation of evidence-based practice skills as an outcome, but as this measure lacked a comparison, it has not been included in Fig. 4 [54].

### Secondary outcomes

#### Confidence

Five randomised trials measured confidence. A total of 13 measures were used, with seven measures from four studies demonstrating statistically significantly better performance in the educational intervention group than in the control group. This included confidence in interpreting medical statistics [39, 40], ease of assessing if a claim is supported by appropriate research [37] and confidence (which was defined as completing a task or performing a particular behaviour in order to realise goals) [48]. Four other measures (from four randomised trials) demonstrated better results from the educational intervention groups than among controls, but the differences were not statistically significant [37, 39–41].

#### Perception of knowledge and/or abilities

No randomised trials assessed perception of knowledge and/or abilities, but it was assessed in two non-randomised studies using 15 measures [55, 57]. Results for nine of these measures were statistically significantly better for the educational intervention groups than for the control groups. This included seven measures of perception of knowledge and/or abilities in research and statistics [55] and two measures of students’ perceived understanding of ‘enduring understandings’ (foundation concepts that the study authors considered relevant to achieving scientific literacy) and scientific literacy [57].

#### Attitude

Three randomised studies assessed attitude, using 12 different measures. One measure showed statistically significantly better results for overall attitude towards searching in the educational intervention group compared with the control group [41]. Six other measures of attitude in this study were in favour of the educational intervention group, but none of the differences was statistically significant. Results from the four measures of attitude in the Nsangi et al. trial 2017a [37] were not statistically significant and favoured neither the educational intervention group nor the control group.

One randomised study, which compared learners’ attitudes towards research and randomised trials, is not included in Fig. 4 as it compared results before and after exposure to different formats of the same information but presents no between-group comparisons [45].

#### Behaviour

Two randomised trials assessed behaviour using six measures. Differences in the three measures were statistically significant in favour of the educational intervention group in one study [37]. Another measure favoured the educational intervention group, but the difference was not statistically significant [48]. The two remaining measures favoured neither group.

#### Satisfaction

Nine randomised trials used a combined total of 27 measures of satisfaction with the educational interventions [37, 39–42, 47–50]. For 14 measures (from three studies), there were statistically significant differences favouring the educational intervention groups [39, 47, 49]. For seven measures (from the same three studies), the differences favoured the educational intervention groups but were not statistically significant. The remaining six measures were not included in Fig. 4 as they either had no control [37, 41, 42, 48, 50] or no participant numbers [49]. Another four measures from three two-group comparison studies [52, 54, 60] assessed satisfaction but are not included in Fig. 4 as they lacked a control comparison.

## Discussion

This review found 24 eligible studies (14 randomised trials and 10 other eligible study types) which had examined the effects of educational interventions about key concepts for assessing claims about health interventions. Measures of the primary outcomes (knowledge, knowledge and skills) were better in educational intervention group participants than in comparison group participants, with statistically significant differences for the majority. The effect of the educational interventions on secondary outcomes (such as confidence, perception of knowledge and/or abilities, attitude, behaviour and satisfaction with the educational intervention) was less consistent. Some studies found statistically significant between-group differences favouring the educational intervention group, some found differences favouring the educational intervention group but which were not statistically significant, while a few reported results which either favoured neither group or favoured the comparison group.

Across the studies, most of the outcomes were measured immediately following the conclusion of the educational intervention, with only a few studies measuring outcomes a short time (typically 2 to 6 weeks) later. Hence, whether the effects of the educational interventions are sustained in the long term is unknown.

Apart from two trials which were reported in the same article [39, 40], none evaluated the same educational intervention as any of the other included studies. Some variation was expected due to the inclusion of different target learners (e.g. children and adults) and different settings (e.g. formal education settings, community groups), but even beyond this, there was substantial variation in the content taught, the intensity and duration of the educational interventions, the educational format and the educational intervention provider.

The situation was similar for outcome measures, with no two studies using the same outcome measures except for the two trials that were performed and reported together [39, 40] and three other trials that were associated [37, 38, 48]. One consequence of this wide range of measures is that synthesis of results from multiple studies is hampered. Few of the measures used were validated, with most developed for each study. A set of flexible evaluation tools (CLAIM Evaluation Tools) that can be used to measure people’s ability to assess claims about treatment effects has recently been developed and validated [62]. This outcome assessment was used within three included recent studies [37, 38, 48]. The use of common outcome measures in future studies of such educational interventions would facilitate comparison of results and synthesis.

Risk of bias in the studies was variable, with the risk high in at least one domain for all randomised studies. There were a few good-quality randomised trials and mostly high or uncertain risk of bias in the other trials. Given the heterogeneity and risk of bias in the studies included in our review, it is difficult to draw firm overall conclusions about the effects the educational interventions on the outcomes of interest. Neither is it possible to make recommendations about the characteristics of educational interventions (such as particular content, duration and format) that are essential. As there are very few head-to-head comparisons of different interventions, currently, there is no reliable evidence that one type of intervention is more effective than another.

That said, educational interventions evaluated in randomised trials with low risk of bias which yielded statistically significant better effects than control and provided a description of educational intervention content and delivery to enable similar projects and potentially easy to implement are promising. Some of these interventions address a wide range of topics, others specific topics, but all of them have only been evaluated in a single trial and need replication.

Promising educational interventions for adults covering a wide range of relevant topics are those evaluated in the trials by Semakula and colleagues [48] and Welch and colleagues [50]. The educational intervention in the Semakula trial was a series of podcasts (which aimed to improve the ability to assess claims about treatment effects; see link in Additional file 1: Table S1). The Welch trial was a web-based module (which covered the basics of the evidence-based process, including literature searching and critical appraisal). Although critical appraisal skills were taught, the Welch study assessed only knowledge, with the impact on skills remaining unknown. A promising educational intervention for adults with a focus on specific topics is the 80-page booklet about understanding disease risk and health intervention benefits and harms (see link in Additional file 1: Table S1 to access) evaluated by Woloshin [39, 40].

For children, promising educational interventions covering a range of topics are those evaluated by Nsangi et al. 2017a [37] and Tait et al. [49] The educational intervention evaluated by Tait [49] was a digitally interactive, individually completed program delivered via an iPad and focused on improving knowledge about clinical trials, including research basics, protocols, randomisation, placebo and blinding. The educational intervention evaluated by Nsangi [37] requires more investment of time (80 min per week over 9 weeks) and used a teacher-delivered program and set of learning resources (including a textbook in comic book format, teacher’s guide, exercises books, poster, activity guides—see link in Additional file 1: Table S1 to access).

To the best of our knowledge, this is the first systematic review designed to identify all the educational interventions developed to improve people’s knowledge and abilities related to the key concepts of critical appraisal of health claims. A strength of the review is our extensive search for and rigorous assessment of the available studies. Our results expand upon the review reported by Nordheim et al. [29], who synthesised similar educational interventions but only those specifically targeted to child learners with school-based educational interventions. Their review included five studies, and the main findings were similar to ours, namely, that the studies showed that educational interventions can improve short-term knowledge and skills (Kirkpatrick level 2), with effects on other outcomes unclear and conclusions limited by the low quality of studies [29]. All but one of the studies in Nordheim’s review were conducted in the USA, whereas there were ten countries represented in the current review, about half of which were conducted in the USA.

### Limitations of study

This review’s limitations mostly arise from the heterogeneity of the included studies and the risk of bias in many of the eligible studies identified. Despite using a comprehensive search strategy, it is possible that some relevant studies have not been identified because of the complex question addressed by our review and also the potential for non-English studies to have been missed if they lacked abstracts/titles in English.

## Conclusion

Considering that people now enjoy increased access to health information and are more involved in making decisions about their health than previous generations, it has become increasingly important that they have the knowledge and skills to assess which information is trustworthy. This review has shown that educational interventions can improve knowledge and skills, at least in the short term, and drawn attention to educational interventions that have been shown to have this effect. The longer-term effects and effects on behaviour, attitudes and confidence remain uncertain.

More certain estimates of the effects of educational interventions on critical assessment of health claims require well-designed educational interventions, validated outcome measures (including measures of skills and longer-term follow-up), rigorous study designs, such as pragmatic randomised trials, and assessment in a variety of populations (considering ethnicity, socioeconomic status and education levels).

## Additional files

Additional file 1: **Table S1.** Characteristics of included studies. (DOCX 69 kb)

Additional file 2: **Table S2.** Results for primary outcomes from included studies (all study designs; randomised trials presented first). (DOCX 60 kb)

Additional file 3: **Table S3.** Results for secondary outcomes from included studies (all study designs; randomised trials presented first). (DOCX 58 kb)

## Abbreviation

ROBINS-I: A Cochrane risk of bias assessment tool: for non-randomised studies of interventions
