# Supplementary material for: Educational interventions to improve people’s understanding of key concepts in assessing the effects of health interventions: a systematic review
Source: Syst Rev. 2018 May 2;7:68. doi: 10.1186/s13643-018-0719-4 (PMC5930693; doi:10.1186/s13643-018-0719-4)
Supplement: Supplementary file 1 — Table S1. Characteristics of included studies. (DOCX 69 kb) [file 13643_2018_719_MOESM1_ESM.docx]

**Table 1 Characteristics of included studies**

| **Author Year; Study type** | **Participants*** | **Setting, Country** | **Intervention details** | | | | **Comparison** |
| --- | --- | --- | --- | --- | --- | --- | --- |
|  |  |  | **Intervention type and content** | **Pedagogical theory** | **Education provider and teaching strategy†** | **When and how much** |  |
| **RANDOMISED STUDIES** | | | | | | | |
| Austvoll-Dahlgren 2012; randomised trial | 96 parents with children aged <4 years  Sampling: convenience Age: NR, Females: 80%, Ethnicity: NR, Education: PS 9%, HS 16%, TE 75% | Web-based module, Norway | Access to a health literacy web-portal (aimed at improving the public’s access to evidence-based health information and health literacy skills) through an email containing the URL. Tasks: 1) access to health research databases, an introduction to research methods, the principles of science and levels of evidence synthesis; 2) a checklist for critically assessing health information (DISCERN) and why critical assessment is important; 3) a checklist for consultations with health care providers and health decision making.  The web portal is described in more detail in another paper [58] or can be viewed online at [www.sunnskepsis.no](http://www.sunnskepsis.no) (website is in Norwegian). | Zarcadoolas’ methods; Central literacy domains: fundamental, science, civic, and cultural. | Individually-completed, interactive online portal, 3 days to explore portal prior to emailed online tasks; 1 task per week for 3 weeks. | Participant controlled; approx. time to complete not reported. | No access to the web portal |
| Barnett 2005; randomised trial | 374 school children Sampling: convenience Age: 9-11 years; Gender: NR; ethnicity: NR | School classroom, UK | Leaflets with three different methods of information presentation, aimed at improving children’s understanding of concepts considered essential for inclusion in adult information forms (eg. randomisation, safety and effectiveness): Group A - Question and answer format, Group B - Story format, Group C - Text format.  Leaflets and questionnaire available from:  <http://adc.bmj.com/content/suppl/2005/03/17/90.4.364.DC1> | Question and answer format, and concepts presented as stories, were hypothesised to lead to a greater understanding of concepts about RCTs than by traditional block text format. | Individually-completed, leaflets. | Participant controlled; approx. time to complete not reported. | Not applicable (as 3 forms of interventions compared) |
| Dunn 2006; randomised trial | 49 people with schizophrenia or schizoaffective disorder  Sampling: convenience Age (mean): 56 years, Females: 45%, Ethnicity: Caucasian 92%, Education (mean): 12.7 y | Not reported specifically (meeting room of some kind), USA | A brief PowerPoint educational module, in the context of consent for hypothetical double-blind placebo-controlled clinical trial.  The four slides were designed to convey more information about the purpose, nature, and risks of placebos in research. The first slide described the placebo both graphically and verbally. The module then described risks of placebos, as well as contextual information that symptoms could get better or worse regardless of which agent people received. | NR | Provider: NR; taught using PowerPoint presentation. | Participant-controlled; approx. time to complete not reported. | A routine explanation of placebos. |
| Hendricks 2001; randomised trial | 220 students in science classes Sampling: convenience Age: NR, Females: 52%, Ethnicity: NR, Education (current): grade 7. Other details: free or reduced-price lunches: 64%, from middle class households: 25% | School classroom, USA | Using situated instruction, students were engaged in a discussion of research, cause-and-effect factors, whether manipulation and control of extraneous factors were used, and shown how to find important pieces of information in actual studies in order to assess causality.  Students were split into groups to examine a magazine containing brief articles to which learning could be applied.  Transfer instruction was provided for all students: students were told their learning could be applied to real world situations and key words and phrases such as "research", "study" and "findings suggest" could help to remind them about causality when making judgments about reports described in newspapers, magazines and news programs. | Situated instruction (active learning) to teach causality; modelling, coaching and scaffolding used, before reducing amount of assistance given. | Grade 7 science teacher provided; school classroom lessons  Teachers (considered to be matched; age and experience) received approximately 5 hr of training in how to use the lesson plans. Instruction began 2 weeks after teacher training had commenced. | Fixed schedule, 4 days; 50 mins per day. | Abstracted instruction (authoritative learning) to teach students causality |
| Kruse 2000; randomised trial | 428 adults (≥18 y); can read and understand Danish; not included in another clinical trial on the same day.  Sampling: convenience Age (mean): 47 y; Females: 63%; Ethnicity: NR, Education level: PS 19%, HS 45%, TE 28%. | Outpatient clinic, Denmark | Three different types of educational materials (varying in length, reading level and reader appeal, degree of detail and presentation style) aimed at improving general understanding of design and conduct of randomised clinical trials: Group A: leaflet (1.5 A4 pages), Group B (2.5 A4 pages): brochure, Group C: booklet (12 pages).  The information materials explained basic aspects of research and RCTs, covering aim, background, formalities, ethics, informed consent, randomization, blinding, and placebo. In addition, the booklet contained paragraphs describing drug development and financial aspects of research. | Based on journalism theories and practical communication experience. | Individually-completed, information item. | Participant controlled; 10-30 mins to complete (depending on the length of the information and personal skills). | No exposure to any of the 3 intervention formats. |
| Ndebele 2012; randomised trial | 36 adults with low understanding of randomisation, double-blinding and placebo Sampling: purposive Age: 20-35 y 93%; all African females; Education level: Grades 5-8 67%, High school 28%. | Meeting room, Africa (Malawi) | Education session using everyday examples used to explain clinical trial procedures (concepts of research, randomisation, double-blinding and placebo) and implications; e.g. farmers given eligibility criteria (including one acre plot and willingness to participate), randomised by picking pieces of paper from a hat, some bags of fertiliser looked exactly like the test fertiliser but did not have any of the chemicals (placebo).  Implemented in a narrative style, in the local language, using a PowerPoint presentation which included vignettes and colourful pictures to supplement written information.  After narration, the presenter related the Irish potato fertiliser research narrative and procedures of the microbicide clinical trial, including trial concepts under study (and their justification and personal implications). | Used a culturally relevant approach to explain clinical trial procedures and their implications. | Provider: NR; mixed methods teaching; group sessions, discussions, presentations. | Fixed single session; duration not reported. | Standard informed consent information and health information on cervical cancer. |
| Nsangi 2017a; randomised trial | 12, 639 year five primary school students from 120 schools in Uganda, cluster randomisation  Sampling: multi-stage stratified random sampling  Age (median): 11 years; Females: 45%; Ethnicity: not formally recorded, but authors advised it that most were Ugandan (A. Nsangi, personal communication, September 28, 2017); Education: grade 5 (current level). | School classroom, Uganda (Sub-Saharan Africa) | Informed Health Choices primary school course, comprising 9 lessons, introduced the idea of claims, treatments and effects and covered 12 key concepts people need to understand when assessing claims about treatments effects, including: fair comparisons of treatments, bad bases for claims, advantages and disadvantages of a treatment. Resources included: textbook, teacher’s guide, exercise books, a poster, activity cards, two day introductory workshop for teachers and a song.  Available from: <http://www.informedhealthchoices.org/primary-school-resources/> | Resources appropriate for primary school learners, including activity-based interactive classroom and home activities using comic book stories, were developed in conjunction with Ugandan primary school teachers and children, and subsequently user-tested, piloted and assessed for feedback incorporated. | Teacher provided; mixed methods teaching. | Fixed schedule, over 9 weeks, 80 mins (one double period) per week. | Assigned to usual curriculum. |
| Nsangi 2017b; randomised trial | 150 (which increased to 159 with an additional 9 intervention teachers) year 5 school teachers from 120 schools in Uganda, cluster randomisation.  Sampling: purposive.  Age: NR; Females: 42%; Ethnicity: not formally recorded, but authors advise most were Ugandan (A. Nsangi, personal communication, September 28, 2017); Education: TE 75%. Other details: Science main subject taught 77%. | Community meeting room, Uganda (Sub-Saharan Africa)Africa (Uganda) | Introductory workshop about Informed Health Choices program (see Nsangi 2017a) [32] The intervention comprised of receiving teachers’ guides (to enable them to familiarise themselves with the content and prepare a plan for delivering the lessons) one week before a two-day introductory workshop to inform of study objectives, train the teachers on the content, ensure understanding of procedures for delivering the content, administering the outcome measure and addressed any questions or concerns. The study participants then delivered nine weeks of the Informed Health Choices program (described in Nsangi 2017a).  Teachers’ guide and IHC program available from: <http://www.informedhealthchoices.org/primary-school-resources/> | Not reported. | Researcher provided; mixed methods teaching. | Mixed schedule, participant controlled for 1 week, intense over 2 days then 80 mins weekly for 9 weeks. | Assigned to usual curriculum. |
| Santesso 2015; randomised trial | 193 patients and members of the public ≥ 16 y. Sampling: convenience Age: 26-65 y 76%; Females: 73%; Ethnicity: NR, Education: some HS or college 40%, diploma or degree 60%. | Web-based module, international  (Canada, Norway, Spain, Argentina and Italy) | New format plain language summary (PLS) presenting a summarised result of a Cochrane systematic review about the effects of a treatment (Vitamin C for preventing and treating the common cold) received via email link. A structured presentation using a question and answer approach and communicating benefits and harms with reference to the magnitude of effect and the quality of evidence.  Two key parts: 1) a narrative summary of the evidence, including a) an introduction to the concept of a systematic review; b) background information about the condition and treatment; and c) information using standardized qualitative statements about the magnitude of the effect and the quality of the evidence for important outcomes (e.g., ‘‘Vitamin C probably decreases how long a cold lasts by a few hours’’); 2) a table presenting the same outcomes and qualitative statements about the effect but also the numerical results.  Supplemental materials available from: <http://www.jclinepi.com/article/S0895-4356(14)00213-3/addons>  Questionnaire available from: <http://www.jclinepi.com/cms/attachment/2035385555/2050851753/mmc1.pdf> | Following principles of linguistic frameworks: based on research and development work over the past 15 years. | Individually-completed, online material. | Participant controlled; approx. time to complete not reported. | A "plain language summary" of a systematic review of a common topic. |
| Semakula 2017; randomised trial | 675 parents of grade five children (fifth year of school) at 35 primary schools in Uganda that were participating in a linked trial**.** [32]  Sampling: convenience, from parents who attended recruitment meetings at schools participating in the linked trials (Nsangi et al 2017a).  Age: NR, Females: 76%, Ethnicity: not formally recorded, but authors advised it is likely that the majority were Ugandan (D. Semakula, personal communication, September 28, 2017); Education: PS or less 50%, HS 30%, TE 20%. | Meeting rooms at schools, Africa (Uganda) | Podcast developed to complement the learning resources developed in the linked trials.[32, 33] Participants could choose to listen to the Informed Health Choices podcast in either English (12%) or Luganda (88%).The podcast comprised 13 audio messages; an introductory episode, eight main episodes of 5-10 minutes (each with at least one Key Concept, a conclusion episode summarising the key messages in each episode of the podcast), and 3 recaps of about 2 minutes, each with a summary of the last two episodes except for the last two which were recapped in the conclusion episode.  The main episodes (designed to entertain and teach) used drama skits to introduce a common claim, the basis of which related to a Key Concept to be taught in that episode.  The trustworthiness of the claim was discussed and assessed, applying the relevant Key Concept, using simple, commonly relatable analogies, explanations and examples. The conclusion to each episode reiterated the main message in the episode and reminding listeners to think critically. The IHC theme song reinforced the key messages in the podcast.  Podcast episodes available from: <http://www.informedhealthchoices.org/podcast-for-parents/> | In consultation with journalists and the general public in Uganda, based on the same Key Concepts list used for the educational intervention for primary school students in the linked trial. [32] | Individually completed, Podcast episodes. | Mixed (fixed and participant controlled components), over 7 to 10 weeks, 20 mins per week. | Listened to a podcast (participants chose language: English or Luganda) containing typical public service announcements about health issues on topics similar to those discussed in the podcast of the intervention group. |
| Tait 2015; randomised trial | 284 parents and children (10-17 y) attending one of paediatric clinics Sampling: convenience Age (mean): parents 41y, children 13y, Females: parents 67%, children 50%, Ethnicity (parents): White 75%, African-American 18%, Education (parents): <HS or some college 56%, >college grad 44%, children current grade (median) 8.  Other details: previous research participation: parents 26%, children 13%. | Outpatient clinic, USA | Interactive program comprising modules that described clinical trial concepts; e.g. participation, protocol, randomisation, placebo, blinding, double-blinding, effectiveness and informed consent. Information presented in visual and written formats together with a narrative ‘voice-over’. Screens were presented sequentially and could not be skipped. Five interactive exercises (e.g. ‘point and touch’ or ‘touch and drag’ on the screen to select their responses, including a jigsaw puzzle solved by matching a term with the correct answer) were included which employed corrective feedback: coloured icons or sound to indicate a correct or wrong answer.  Author advises (A. Tait, personal communication, September 20, 2017): there is no further detail about intervention, other than that described in the paper. The technology used was proprietary. Our group at Michigan was simply charged with testing the intervention; we had no part in the development of the intervention other than to suggest content. We have no commercial relationship with the company. | Computer-visualisations (using two-dimensional and three-dimensional graphics software) described the clinical trial concepts. | Individually-completed, digital program on iPad, tailored to 7–8th grade audience. | Participant controlled; approx. time to complete not reported. | Paper version of the text only (no exercises included). Tailored to 7th–8th grade audience. |
| Welch 2014; randomised trial | 473 members of the National Athletic Trainers Association Sampling: convenience Age (range of mean ages): 23-41 y, Females: 55%, Ethnicity: NR, Education: NR.  Previous evidence-based instruction: 47% | Web-based module, USA | Ten modules developed to improve understanding of the "evidence-based" process, received via email link:  Steps of Evidence Based Practice and Module Topics  Step 1 Developing a clinical question  - Module 1 Developing clinical questions  Step 2 Literature searching  - Module 2 Literature searching  - Module 3 Types of research  Step 3 Critical appraisal  - Module 4 Levels of evidence and strength of recommendation  - Module 5 Appraisal scales  - Module 6 Statistics terminology  - Module 7 Reliability coefficients  - Module 8 Critically appraised papers and topics  Step 4 Applying the evidence  - Module 9 Patient-reported outcomes  - Module 10 Disablement models | Providing the opportunity to complete material at participants' own speed and convenience. | Individually-completed, online program. | Flexible access, over 4 weeks, 10 modules each taking ~20-25 mins to complete. | No exposure to intervention. |
| Woloshin 2007a; randomised trial | 334 high socioeconomic status adults  Sampling: convenience Age (median): 61 y; Females: 81%, Ethnicity: White 99%, Education: college or university ~75%.  Household income $50-99,000 48%. | University lecture hall/home‡, USA | Booklet (~80 pages) to improve understanding of disease risk and how to understand the benefits and harms of interventions (title: Know Your Chances: Understanding Health Statistics).  Participants received the primer booklet in the mail. The first part of the primer teaches people how to understand disease risk by using the example of colon cancer. The second part focuses on how to understand the benefits and harms of interventions by using a Zocor (high cholesterol treatment) direct-to-consumer advertisement for secondary heart disease prevention.  Available from: <https://www.ncbi.nlm.nih.gov/books/NBK115435/> | Designed to be inviting and nonthreatening; use of cartoons and figures, and quizzes (with answers). | Individually-completed, booklet, tailored to ≤8th grade audience. | Participant controlled, over 2 weeks; approx. time to complete not reported. | Booklet, similar length and reading level, with general risk information. It did not include training on how to interpret quantitative information. |
| Woloshin 2007b; randomised trial | 221 low socioeconomic status adults  Sampling: convenience Age (median): 58 y; Females: 20%, Ethnicity: white 92%, Education: HS or college degree ~80%; Household income: $25-49,000 34%. | Outpatient clinic/home‡, USA | Same as Woloshin 2007a | Same as Woloshin 2007a | Same as Woloshin 2007a | Same as Woloshin 2007a | Same as Woloshin 2007a |
| **OTHER STUDY DESIGNS** | | | | | | | |
| Berger 2010; Controlled study with only post-test measures. | 204 professional counsellors, self-help group members, and professional patient advocates Sampling: convenience Age: NR, Females: 79%, Ethnicity: NR, Education: higher education degree 71%. | University classroom, Germany | Workshop to improve basic Evidence-Based Medicine (EBM) competencies, including how to: 1) ask a question that can be answered, 2) identify appropriate sources and perform a systematic literature search, 3) critical appraisal, 4) communicating results. Also covered: a) basic statistics, b) consumer information and the media, c) risk communication, d) clinical testing of new drugs, e) the role of patient representatives.  A handbook of about 60 pages was used, comprising of publications, vocabularies, glossaries, work sheets and supplemental information (e.g. lectures, worksheets, flip-charts, videos, study abstracts and tables, critical appraisal sheets, mind-maps, handouts, articles, information sheets, library databases).  Various topics of controversy in medicine and health care were used to demonstrate the relevance and general principles of EBM;  - Hormone therapy in (post-) menopausal women  - Screening for colorectal cancer  - A critical appraisal of the meta-analysis on the effects of homeopathy  Curriculum available from:  <http://www.biomedcentral.com/content/supplementary/1472-6920-10-16-S1.doc>  Examples of successful implementation (as reported by participants) available from:  <http://www.biomedcentral.com/content/supplementary/1472-6920-10-16-S2.doc> | EBM, theoretical framework of critical-constructive teaching; Klafki’s method. | Research team provided workshop, mixed teaching methods: small group work, presentations and individual work. | Fixed, 1 week long; intensity: NR. | A university EBM course of comparable content and methods (no further details provided). |
| Ciarocco 2013; Controlled study with only post-test measures. | 83 university students enrolled in a research methods course Sampling: convenience Age (mean): 23y; Females: 76%, Ethnicity: NR, Education (current level): university undergraduates. | University classroom, USA | Research methods course to teach five specific experimental designs and increase real-life application for research concepts: answering a research question, describing and critiquing study methods and results, the two-group design, variables, procedure, and statistical analyses of the research, critiques of the studies’ strengths and weakness.  Activities: an in-class demonstration of the research design with students role-playing as research participants; working in small groups to implement a study with a specific research question, working hypothesis, relevant variables defined, data collection (from a small convenience sample) and analysis before reporting the results. | Active learning strategies; instructional scaffolding to promote understanding and development. | University lecturer provided, mixed teaching methods: interactive sessions, small groups, demonstrations and guided learning. | NR | Similar information covered, however, specific information and skills were not covered (e.g. collecting, analysing or reporting research findings). |
| Derry 1998; non-randomised trial with concurrent controls. | Unknown total of students in eighth grade Sampling: convenience Age: NR, Females: NR, Ethnicity: "diverse", Education (current level): 8th grade. | School classroom, USA | Students watched and discussed (with researchers and teachers) a popular movie on laser disc, Lorenzo's Oil; an emotional drama focusing on ethics in medical research, experimental control, informed single-case observations versus randomized clinical trials, and governmental regulation of the scientific and lay community.  Presentations were given on: social issues depicted in the film, credible scientific research, valid statistical inference; and thinking as evidential argument.  Activity: a lengthy mock legislative hearing dealing with government regulation of the dietary supplement and vitamin industry (an issue that shared several story line parallels with Lorenzo’s Oil) with small groups preparing arguments for those with vested interests (members of the Food and Drug Administration, and vitamin manufacturers etc.), or representing more balanced positions (journalists, state legislators etc.). | Based on Vygotsky, Dewey and radical constructivism): understanding through interaction. | Teacher provided, mixed teaching methods: interactive and didactic, with group discussions.  Eighth grade teachers. Each had previously participated in workshops on mentoring and statistical reasoning in preparation for these roles. | Fixed, 3 weeks; 70 minute sessions daily. | Usual teaching |
| Kaelin 2007; non-randomised trial with concurrent controls. | 1465 students Sampling: convenience Age (mean): 12.2y, Females: 53%, Ethnicity: 54% Hispanic, 37% African American, 10% Caucasian, 2% Asian/Pacific, Education (current level): Grade 7. | School classroom, USA | Middle school epidemiology curriculum called Detectives in the Classroom, was developed to present epidemiology as the science of public health, using health-related issues that capture the interest of young students and help prepare them to make evidence-based health-related decisions.  Comprising 34 lessons, in five modules, each focused on one of five epidemiologic essential questions and its enduring understanding, aligning respectively with the following content: 1) use of descriptive information to generate hypotheses; 2) use of analytic techniques and study designs to investigate associations; 3) evaluation of causality; 4) role of epidemiology in societal decisions about risk and prevention; and 5) assessment of prevention strategy effectiveness. The lessons were shaped to help students “uncover” the epidemiologic principles and apply what they learn to health issues of interest in their personal and public lives. | Based on “essential questions” to develop “enduring understandings”, pedagogical principles suggested in Understanding by Design. | Seventh-grade teacher provided (either health, science, and / or mathematics educators - actual expertise not specified); school classroom lessons.  Teachers attended five preparation workshops. The lessons were taught before the next workshop and teachers shared their experiences. | Fixed schedule, school year; 34 lessons in 5 modules. | Usual teaching |
| Leshowitz 2002; controlled before and after study. | 226 university students enrolled in research methods class Sampling: convenience Age: NR, Females: NR, Ethnicity: NR, Education (current level): university students and academic tutors (all mostly juniors). | University classroom, USA | Methodological reasoning course involving discussion and analysis of a problem: no lecture and no text-book. Students prepared by reading several articles from the popular media and completed several worksheets that explored various facets of the topic. The instructor integrated the principles of methodological reasoning directly into the analysis of information bearing on the problem. The lessons often challenged the unquestioned opinions and beliefs of students on current societal issues.  For example: students would critically analyse the information in an article, and re-evaluate the basis of their beliefs in a reflective dialogue: What data were collected in the study presented in the article? How was the study carried out? Do the data show an association between, for example, divorce and psychological symptoms? Could other factors besides, for example, the divorce of their parents have contributed to the purported behavioural problems? Was the journalist unbiased in the collection of statistics and testimonies of experts? Does the presence of inflammatory language indicate a possible agenda of the journalist? Summarizing the critical analysis, the instructor asked the students: What do you know about this issue and how do you know it? | A course in effective thinking that meets the university’s general studies requirement in critical inquiry. | Provider: NR; interactive teaching methods: discussion and analysis of a problem. | NR | Usual teaching in a similar course |
| Leshowitz 1993; controlled study with only post-test measures. | 55 students in special (intervention) and general (comparison) education classes.  Sampling: convenience Age: 16–20 y; Females: 32%, Ethnicity: 68%; Caucasian, 32% Hispanic, Education (current level): Grades 7 - 12. | School classroom, USA | Critical thinking and scientific reasoning course developed for school students with learning disabilities designed to teach the ability to analyse data to establish a causal relationship between variables, and understanding of the principles of scientific reasoning, through:  1) evaluation of advertisements - a review of fact and opinion;  Students were asked read a newspaper article and state the underlying question or problem. The teacher then guided them through a discussion of what was found in the study and how the author answered the underlying question, including analysing the information within graphic representations of data. Effort was devoted to identifying the independent and dependent variables and plotting their relationships in X-Y plots.  2) evaluation of studies - stating the bottom line conclusion reached, and whether the data supported the conclusion;  (a) Is there a relationship between the antecedent condition (independent variable) and the effect (dependent variable)? (b) Does the independent variable precede the dependent variable in time? and (c) Can the effect be attributed to the independent variable? Alternatively, is the influence of extraneous "third" variables or "rival hypotheses" controlled or removed in the study by the use of a control group? | Multiple approaches: Grossen and Gamine's (1990), Algozzine (1986), Darch and Kameenui (1987).  Instructional approach was Socratic dialogue: to actively engage the students and inspire reflective consideration of information and to foster a learning environment of critical inquiry. | Research team provided, Socratic dialogue format, one teacher per class, class sizes ranging from 3-10.  Intervention presented by the second and third authors Karen Jenkens and Shauna Heaton, who are special education teachers. | Fixed, 4-6 weeks; 40 minute sessions daily. | Usual teaching in general education classes |
| Matic-Strametz 2013; controlled before and after study. | 87 university students in training to become biology teachers  Sampling: convenience Age: NR, Females: NR, Ethnicity: NR, Education (current level): university undergraduates. | University classroom, Germany | Human biology course providing education in the fundamentals of EBM and good scientific methodology (GSP): students were asked whether probiotic yoghurt drinks have a positive impact on the human immune system, since the promotion of this product and consumer organizations media attention came to very different opinions. The focus was on the formulation of scientific questions and the evaluation of interventional studies.  14 days after the intervention, students worked in small groups to select a topic and independently prepare an hour long problem-based teaching unit, which they then delivered to students in grades 8 and 9. | NR | Provider not reported, block seminar. | Fixed, 2- 4 days; total of 24 hours. | Usual teaching |
| Ouimet 2015; controlled before and after study. | 29 Political Science Masters students Sampling: convenience Age: NR, Females: 54%, Ethnicity: NR.  Exposure to quantitative research methodology: completed courses Undergrad 65% Graduate 38%, currently enrolled 19%. | University classroom, Canada | Science and policy analysis course covering the different types of research questions and hypotheses; how to develop a search strategy and systematically find research studies; the different types of quantitative and qualitative research designs and critical appraisal tools.  Students individually critically appraised one systematic review, one randomised controlled trial, one quantitative observational study and one qualitative study using an adapted critical appraisal tool embedded in a web-based review software (EPPI-Centre at the Social Science Research Unit of the Institute of Education, University of London).  Activity: small groups conducted a streamlined version of a systematic review of prospective experimental or quasi-experimental primary studies on a research question of their choice. Each team had to extract the data from only six primary studies, regardless of the number of studies that passed the inclusion and exclusion criteria. Students gave oral presentations of the findings of the shortened systematic review produced by each team. | NR | University lecturer provided, mixed teaching methods; didactic and small group work. | Fixed, 16 weeks; 2hrs 50 mins per week. | A standard research methods course |
| Rowe 2015; controlled before and after study. | 475 (outcome 1) and 1443 (outcome 2) university science students Sampling: retrospective and convenience Age (mean)§: 22 y, Females: NR, Ethnicity§: White 57%, Hispanic 18%, African-American 17%. | University classroom, USA | General education science course that used discussions and PowerPoints along with tasks and activities, to teach the importance of evidence, skepticism, and the need for multiple working hypotheses when seeking causal explanations. Topics selected to pique non–science student's interest and present the course curricula within an enthusiastic learning environment and included astrology, homeopathy, Bigfoot and intelligent design.  The systematic and objective nature of science was discussed, along with reliable means of evaluating testable claims and strengths and successes of the scientific approach: including its unique reliance on evidence, skepticism, logic, multiple working hypotheses, and Occam’s razor (the foundations of science). The importance of self-correction was emphasised, and the pernicious effects of dissonance, dishonesty, and bias are introduced, as impediments to understanding. The limits to perception and memory are discussed, including why anecdotal evidence, eyewitness accounts, and even personal experiences are insufficient for accepting a claim.  Two textbooks and a lab manual used:  1) Foundations of Science - Custom (This is a custom edition of Conceptual 690 Integrated Science) [59]  2) How to Think About Weird Things: Critical Thinking for a New Age (paperback) [60]  3) Lab manual: Foundations of Science Lab Manual ISBN 698  Supplemental materials: <http://www.lifescied.org/content/suppl/2015/07/24/14.3.ar30.DC1.html> | An operational approach provided by Bernstein et al. (2006). | University lecturer provided, mixed teaching methods: lectures, case studies, group work, class discussions homework assignments. | Fixed, one semester; 2 x 80 minute sessions per week. | Traditional introductory science courses |
| Steckelberg 2009; controlled study with only post-test measures. | 263 secondary school students (grade 11). Sampling: convenience Age (mean): 18 y; Females: 62%,82% had German as first language. | School classroom, Germany | Sessions aimed at improving students' critical health literacy, covering topics such as: misinterpretations of data, study types, critical appraisal and systematic reviews.  Workbook: contained the six modules (comprising 22 lessons) and corresponding worksheets, original publications and a glossary, with a total of 167 pages:  Module 1: Fallacies and misinterpretations of data representation: observational studies versus randomized controlled trials (RCT) – What are the differences?  Module 2: Critical appraisal of RCT’s  Module 3: Informed choice in diagnostic tests  Module 4: Understanding systematic reviews  Module 5: Searching the Internet and databases  Module 6: Appraising Patient Information  Topics: vitamin substitution and smoking (covering nutrition, therapy and smoking), depression screening (covering diagnosis and prevention) and diagnosis in sports injuries (covering diagnosis, sports and fitness and injuries in sports).  Activities: small groups planned and executed projects focusing on the application of specific knowledge or skills and on improving involvement and motivation, in order to foster independent thinking, self-confidence, and social responsibility; students critically appraised topics, such as information on Vitamin A (betacarotin) substitution for smokers and the efficacy of a probiotic drinking yogurt within the project lessons. | Klafki’s five questions, which promote systematic reflection regarding aims and intentions of instruction. | Research team provided, mixed teaching methods: lectures, discussions, small group discussion, worksheets, flip charts, posters, Metaplan25 and computer projections. | Fixed, daily sessions, 5 days; total of 20-24 hours. | Usual teaching |

Abbreviations: NR (not reported); NA (not applicable); PS (primary school); HS (high school); TE (tertiary education).

*Sampling, sample size, age, gender, ethnicity, education

†Assume "face-to-face" unless otherwise indicated

‡Site of recruitment/site of intervention

§ Based on university statistics: the demographic makeup of the course likely represents that of the university
