# Supplementary material for: Educational interventions to improve people’s understanding of key concepts in assessing the effects of health interventions: a systematic review
Source: Syst Rev. 2018 May 2;7:68. doi: 10.1186/s13643-018-0719-4 (PMC5930693; doi:10.1186/s13643-018-0719-4)
Supplement: Supplementary file 2 — Table S2. Results for primary outcomes from included studies (all study designs; randomised trials presented first). (DOCX 60 kb) [file 13643_2018_719_MOESM2_ESM.docx]

**Table 2 Results for primary outcomes from included studies** (all study designs; randomised trials presented first)

| **Author Year; (participant age group)** | **Outcome(s) measured** | **Measure/s used** | **Pre and/or post-test*** | **Numbers analysed** Total n (%) (intervention n, comparison n) | **Results** | | | | | | | | | | | | | | | | | | |  |
| --- | --- | --- | --- | --- | --- | --- | --- | --- | --- | --- | --- | --- | --- | --- | --- | --- | --- | --- | --- | --- | --- | --- | --- | --- |
| **RANDOMISED TRIALS** | | | | | | | | | | | | | | | | | | | | | | | |  |
| Nsangi 2017a  (children) | Knowledge and skills | 24 MCQs, 2 questions (2 and 4 response options) for each of 12 key concepts, taken from the Claim Evaluation Tools† database.  Score: 1 per correct question. Passing score ≥ 13/24. | Post-test | 10183 (81%) (n=5753, n=4430) | Intervention vs comparison  Mean score (SD)  62.4% (18.8) vs 43 .1% (15.2)  Adjusted mean difference: 20.0%, 95% CI 17.3–22.7  Children with a predetermined passing score (≥13 of 24 correct answers)  n (%)  3967 (69%) vs 1186 (27%)  Adjusted difference in proportions: 50%, 95% CI 44–55; p<0.00001.  Children with a predetermined mastery score (≥20 of 24 correct answers)  n (%)  1070 (19%) vs 38 (1%)  Adjusted difference in proportions: 10%, 95% CI 18–18; p<0.00001. | | | | | | | | | | | | | | | | | | |  |
| Nsangi 2017b (adults) | Knowledge and skills | 24 MCQs, 2 questions (2 and 4 response options) for each of 12 key concepts, taken from the Claim Evaluation Tools† database.  Score: 1 per correct question, converted into a percentage. Passing score ≥ 13/24. | Post-test | 152 (96%) (n=85, n=67) | Intervention vs comparison  Mean score (SD)  85% (17.1) vs 67% (14.3)  Adjusted mean difference: 18.3%, 95% CI 12.9–23.3  Teachers with a predetermined passing score (≥13 of 24 correct answers)  n (%),  83 (98%) vs 58 (87%)  Adjusted difference in proportions: 11%, 95% CI 4–13; 7.2 (1.5–35.3); p<0.00001.  Teachers with a predetermined mastery score (≥20 of 24 correct answers)  61 (72%) vs 10 (15%)  Adjusted difference in proportions: 57%, 95% CI 37-70; 14.4 (6.2-33.1); p<0.00001. | | | | | | | | | | | | | | | | | | |  |
| Semakula 2017  (adults) | Knowledge and skills | 18 MCQ, 2 questions (2 and 4 response options) understanding and application of each of 9 key concepts, taken from the Claim Evaluation Tools† database.  Score: 1 per correct question. Passing score ≥ 11/18. | Post-test | 561 (83%) (n=288, n=273) | Intervention vs comparison  Mean score (SD)  67.8% (19.6) vs 52.4% (17.6)  Adjusted mean difference: 15•5%, 95% CI 12.5–18.6; p<0.0001.  Parents with a predetermined passing score (≥11 of 18 correct answers)  n (%)  203 (71%) vs 103 (38%)  Adjusted difference in proportions: 34%, 95% CI 26–41; p<0.0001. | | | | | | | | | | | | | | | | | | |  |
| Woloshin 2007a  (adults) | Knowledge and skills | Ability to interpret medical statistics† (18-item test sent to participants along with interventions, to be completed and returned within 2 weeks).  Score: 0 to 100 scales, higher scores represent higher interest or confidence. | Post-test | 322 (96%) (n=153, n=169) | Intervention vs comparison | | | | | | | | | | | | | | | | | | |  |
|  |  |  |  |  |  | | **Mean scores** | | | | **Difference** | | | | | | | **95% CI** | | | | **P value** | |  |
|  |  |  |  |  | Overall | | 81 vs 75 | | | | 6 | | | | | | | 3-9 | | | | 0.0006 | |  |
|  |  |  |  |  |  | | **% achieving scores** | | | | **Difference** | | | | | | | **95% CI** | | | | **P value** | |  |
|  |  |  |  |  | Score ≥90 | | 26 vs 7 | | | | 13 | | | | | | | 11-27 | | | | <0.001 | |  |
|  |  |  |  |  | Score ≥75 | | 74 vs 56 | | | | 18 | | | | | | | 8-28 | | | | 0.001 | |  |
|  |  |  |  |  | Additional analyses (adjusted for age, sex, level of education, medical conditions) | | | | | | | | | | | | | | | | | | |  |
|  |  |  |  |  |  | |  | | | | 7 | | | | | | |  | | | | < 0.001 | |  |
| Woloshin 2007b  (adults) | Knowledge and skills | Ability to interpret medical statistics† (18-item test sent to participants along with interventions, to be completed and returned within 2 weeks).  Score: 0 to 100 scales, higher scores represent higher interest or confidence. | Post-test | 200 (91%) (n=98, n=102) | Intervention vs comparison | | | | | | | | | | | | | | | | | | |  |
|  |  |  |  |  |  | | **Mean scores** | | | | **Difference** | | | | | | | **95% CI** | | | | **P value** | |  |
|  |  |  |  |  | Overall | | 69 vs 62 | | | | 7 | | | | | | | 2-12 | | | | 0.008 | |  |
|  |  |  |  |  |  | | **% achieving scores** | | | | **Difference** | | | | | | | **95% CI** | | | | **P value** | |  |
|  |  |  |  |  | Score ≥90 | | 10% vs 2% | | | | 8 | | | | | | | 2-14 | | | | 0.014 | |  |
|  |  |  |  |  | Score ≥75 | | 44% vs 26% | | | | 18 | | | | | | | 5 - 31 | | | | 0.010 | |  |
|  |  |  |  |  | Additional analyses (adjusted for age, sex, level of education, medical conditions): | | | | | | | | | | | | | | | | | | |  |
|  |  |  |  |  |  | |  | | | | 6 | | |  | | | | | | | | 0.019 | |  |
| Santesso 2015  (adults) | Knowledge | Understanding of benefits, harms and quality of evidence (5 MCQs) | Post-test | 143 (74 %) (n=74, n=69) |  | | | | | | | | | | **Intervention vs comparison %** | | | | | **Difference** | | **P value** | |  |
|  |  |  |  |  | 1. Overall | | | | | | | | | | 53% vs 18% | | | | | 35 | | <0.001 | |  |
|  |  |  |  |  | 2. Understanding of quality of evidence | | | | | | | | | | 43% vs 2% | | | | | 41 | | <0.001 | |  |
|  |  |  |  |  | 3. Understanding of quality of evidence and risk (qualitative statements) | | | | | | | | | | 47% vs 40% | | | | | 7 | | 0.42 | |  |
|  |  |  |  |  | 4. Ability to quantify risk (dichotomous outcome) | | | | | | | | | | 64% vs 17% | | | | | 47 | | <0.001 | |  |
|  |  |  |  |  | 5. Understanding of risk | | | | | | | | | | 45% vs 19% | | | | | 26 | | 0.001 | |  |
|  |  |  |  |  | 6. Ability to quantify risk (continuous outcome) | | | | | | | | | | 65% vs 10% | | | | | 55 | | <0.001 | |  |
|  | Knowledge | Comprehension of purpose of study and ability to identify producer (2 MCQs)  Score: 1 point per question. The proportion of people who correctly answered a question was averaged over the five total questions and then compared between formats | Post-test | 143 (74 %) (n=74, n=69) |  | | | | | | | | | | **Intervention vs comparison %** | | | | | **Difference** | | **P value** | |  |
|  |  |  |  |  | 1. Comprehension of purpose of summary | | | | | | | | | | 32% vs 45% | | | | | 13 | | 0.17 | |  |
|  |  |  |  |  | 2. Ability to identify producer of review | | | | | | | | | | 89% vs 67% | | | | | 22 | | 0.001 | |  |
| Austvoll-Dahlgren 2012  (adults) | Knowledge and skills | Critical appraisal skills† (rating quality of material). Score: range 1-5: 1–2 = low quality, 3 = moderate quality; 4–5 = high quality, which was measured against ratings made by two blinded external experts. | Post-test | 66 (69%) (n=27, n=39) | Intervention vs comparison Mean rating (SD) 2.4 (0.8) vs 2.4 (1.0), mean difference: 0, p=0.904 Difference between the expert rating (rated as 1) and study groups:  1.4 vs 1.4, difference = 0, p = 0.904 The distribution of the ratings across the two groups: p=0.448 | | | | | | | | | | | | | | | | | | |  |
|  | Knowledge and skills | Ability to identify information based on research. Score: number identified | Post-test | 67 (70%) (n=28, n=39) | Intervention vs comparison  n 2 vs 1, relative risk: 2.8 (CI 0.3–29.2) p=0.39 | | | | | | | | | | | | | | | | | | |  |
| Tait 2015 | Knowledge | Understanding of basic research concepts (9 questions).  Score: 3-point scale of ‘no understanding’ (0), ‘partial understanding’ (1) and ‘complete understanding’ (2).  Range: 0–18 | Pre- and Post- test | 283 (100%) (n=140, n=143) Parents: 148 (n=73, n=75) Children: 135 (n=67, n=68) | Intervention vs comparison (Change Pre-Post) | | | | | | | | | | | | | | | | | | |  |
|  |  |  |  |  |  | **Mean (SD)** | | | | | | **Mean diff** | | | | | | | **95%CI** | | | **P value** | |  |
|  |  |  |  |  | Children | 7.8 (3.1) vs 4.9 (3.4) | | | | | | 3.04 | | | | | | | 1.8 to 4.1 | | | p<0.001 | |  |
|  |  |  |  |  | Parents | 5.4 (3.5) vs 5.0 (3.3) | | | | | | 0.39 | | | | | | | −0.7 to 1.5 | | | not reported | |  |
|  |  |  |  |  | Intervention (post) vs comparison (post) | | | | | | | | | | | | | | | | | | |  |
|  |  |  |  |  |  | **Mean (SD)** | | | | | | **Mean diff** | | | | | | | **95%CI** | | | **P value** | |  |
|  |  |  |  |  | Children | 11.7 (4.1) vs 8.9 (4.1) | | | | | | 2.8 | | | | | | | 1.4 to 4.2 | | | <0.001 | |  |
|  |  |  |  |  | Parents | 13.3 (3.6) vs 12.7 (4.0) | | | | | | 0.6 | | | | | | | −0.6 to 1.9 | | | not reported | |  |
|  | Knowledge | Knowledge of clinical trial participation (10 questions)  Range: 0–20 | Pre- and Post- test | 283 (100%) (n=140, n=143) Parents: 148 (n=73, n=75) Children: 135 (n=67, n=68) | Intervention vs comparison | | | | | | | | | | | | | | | | | | |  |
|  |  |  |  |  |  | **Mean (SD)** | | | | | | **Mean diff** | | | | | | | **95%CI** | | | **P value** | |  |
|  |  |  |  |  | Children | 15.9 (±2.9) vs 12.4 (±2.8) | | | | | | 3.5 | | | | | | | 2.4 to 4.6 | | | <0.001 | |  |
|  |  |  |  |  | Parents | 17.4 (±2.6) vs 16.7 (±2.4) | | | | | | 0.72 | | | | | | | −1.5 to 1.6 | | | not reported | |  |
| Welch 2014  (children and adults) | Knowledge | Knowledge of evidence-based practice† (web-based survey to be completed in 7 days, without the use of external resources: 60 MCQ).  Score: 1 point for each correct response | Pre- and Post-test | 164 (35%) (n=82, n=82) | Intervention vs comparison Total mean difference  +5.70 vs +0.87  Post assessment means (SD)  36.4 (8.6) vs 31 (6.3); p=0 .01  Pre vs Post: mean (SD)  Intervention: 30.7 (5.9) vs 36.4 (8.6), p=0.001  Comparison: 30.1 (5.7) vs 31.0 (6.3), p=0.08 | | | | | | | | | | | | | | | | | | |  |
| Ndebele 2012  (adults) | Knowledge | Including multiple measures, including understanding of  1. Randomisation  2. Double-blinding  3. Placebo  4. Implications  and  5. Composite score  Structured individual interviews.  Score: not stated | Pre- and Post-test | 36 (100%) (n=18, n=18) | **Outcome** | | | | **Intervention Pre vs Post (mean, SD)** | | | | **Control Pre vs Post (mean, SD)** | | | | | | **Between-group difference in change score** | | | **P value** | |  |
|  |  |  |  |  | 1. Randomisation | | | | 76.7 (13.6) vs 91.6 (13.1) | | | | 79.3 (12.2) vs 64.6 (29.2) | | | | | | 29.7 | | | 0 .075 | |  |
|  |  |  |  |  | 2. Double-blinding | | | | 57.8 (22.6) vs 74.1 (19.6) | | | | 54.4 (20.4) vs 44.5 (34.1) | | | | | | 26.3 | | | 0.001 | |  |
|  |  |  |  |  | 3. Placebo | | | | 74.9 (15.4 ) vs 85.2 (26.2) | | | | 74.8 (20.4) vs 65.7 (21.8) | | | | | | 19.4 | | | 0.003 | |  |
|  |  |  |  |  | 4. Implications | | | | 44.1 (16.4) vs 84.7 (12.4) | | | | 41.8 (10.0) vs 42.2 (13.4) | | | | | | 40.2 | | | 0.000 | |  |
|  |  |  |  |  | 5. Composite score | | | | 60.7 (7.9) vs 92.2 (15.6) | | | | 60.4 (9.3) vs 55.7 (10.5) | | | | | | 36.2 | | | 0.0001 | |  |
|  |  |  |  |  |  | | | Intervention vs comparison  Distribution: % change (Pre vs Post) | | | | | | | | | | | | | | | |  |
|  |  |  |  |  | Score 0-49% | | | | -5.6 vs +16.7 | | | | | | | | | | | | | | |  |
|  |  |  |  |  | Score 50-74% | | | | -66.6 vs -16.7 | | | | | | | | | | | | | | |  |
|  |  |  |  |  | Score ≥75% | | | | +13 vs 0 | | | | | | | | | | | | | | |  |
| Kruse 2000  (adults) | Knowledge | Knowledge about clinical research† focusing on randomised trials (17 MCQ questions).  Score: 1 point per correct answer; maximum score = 17 | Pre- and Post-test (2 weeks post intervention) | Total % (Exp. Gr. 1, Exp. Gr. 2, Exp. Gr. 3, Control Gr.) 369 (86%) (n=94, n=96, n=90, n=89) | **Measures** | | | | **Pre vs Post means (SD)** | | | | **Change in mean score (SE)** | | | | | | **Increase in acceptable scores** (at follow-up) % At entry, 46% of the patients obtained an acceptable score. | | | **P value** (compared to control group) | |  |
|  |  |  |  |  | 1. Leaflet | | | | 7.9 (3.1) vs 8.8 (3.4) | | | | 1.0 (0.3) | | | | | | 13% | | | 0.15 | |  |
|  |  |  |  |  | 2. Brochure | | | | 7.8 (3.1) vs 9.5 (3.3) | | | | 1.6 (0.3) | | | | | | 24% | | | < 0.001 | |  |
|  |  |  |  |  | 3. Booklet | | | | 7.9 (3.1) vs 9.3 (3.1) | | | | 1.4 (0.3) | | | | | | 8% | | | 0.007 | |  |
|  |  |  |  |  | 4. Control | | | | 8.2 (2.9) vs 8.7 (2.7) | | | | 0.5 (0.2) | | | | | | 7% | | | reference | |  |
| Dunn 2006  (adults) | Knowledge | Understanding placebos† (12-item questionnaire, comprising 6 closed-ended and 6 open-ended questions examined demographic, clinical, neurocognitive, and decision-making correlates of understanding of placebos).  Score: 0 (incorrect) or 1 (correct); max. score of 12 | Post-test | 49 (100%) (n=24, n=25) | Intervention vs comparison  Mean (SD), difference, p value.  8.2 (2.7) vs 5.8 (2.5); 2.4, p = 0.002  True/false items only:  5.0 (1.1) vs 3.7 (1.5), 1.3, p = 0.001 | | | | | | | | | | | | | | | | | | |  |
| Barnett 2005  (children) | Knowledge | Understanding  1. Randomisation  2. Concepts related to safety and effectiveness  12 item questionnaire with yes / no / don’t know response. Score: n (%) who got all three correct for each section | Post-test | 342 (91%) (n=115, n=110, n=117)  Total (A, B, C) |  | | | | | | | A vs B vs C | | | | | | | | | | |  |  |
|  |  |  |  |  | **Measure** | | | | | | | **Q and A format** | | | | **Story format** | | | | **Text format** | | | **P value** |  |
|  |  |  |  |  | Randomisation | | | | | | | 8 (7%) | | | | 23 (21%) | | | | 15 (13%) | | | <0.001 |  |
|  |  |  |  |  | Concepts related to safety and effectiveness | | | | | | | 8 (7%) | | | | 36 (33%) | | | | 17 (15%) | | | <0.0001 |  |
| Hendricks 2001  (children) | Knowledge and skills | Ability to apply the principle of causality† (test of objective questions and scenarios)  Score: range 0-15, converted to percentage scores (higher result demonstrating increased abilities). | Post-test | 220 (100%) (n= 115, n=105) | Intervention vs comparison  Mean scores (SD)  84.6 (17.9) vs 69.9 (19.8); mean difference: 14.4 (99% CI: 8.1 to 21.3) | | | | | | | | | | | | | | | | | | |  |
|  | Knowledge | Conceptual understanding of causality† (open-ended questions).  Score: responses coded as correct if they included explanation of the need for experimental manipulation and control of nuisance factors to show cause-effect relationships. | Post-test | 135 (61%) (n=88, n=47) [only students with scores ≥ 80% on post-test were included in this analysis] | Intervention vs comparison  n (%)  68 (77) vs 12 (26), (95% CI: 0.37 – 0.67) | | | | | | | | | | | | | | | | | | |  |
| **OTHER STUDY DESIGNS** | | | | | | | | | | | | | | | | | | | | | | | |  |
| Kaelin 2007  (adolescents) | Knowledge | Ability for epidemiological reasoning† (11 MCQ items from a 62 item test). Score: No further details reported. Exp. Gr. 1: more experienced teacher and 16 lessons taught,  Exp. Gr. 2: less experienced teachers and 16-18 lessons taught,  Exp. Gr. 3: less experienced teachers and 6–10 lessons taught.  Cont. Gr. 1: students whose teacher was assigned to control,  Cont. Gr. 2: additionally recruited "non-volunteer” teachers and their students. | Pre- and Post-test | 997 (68 %) | Intervention vs comparison  (Exp. Gr. 1 vs Exp. Gr. 2 vs Exp. Gr. 3) vs (Cont. Gr. 1 vs Cont. Gr. 2) Analysis of covariance (ANCOVA): Least squares means (LS), accounting for differences in pre-test scores and other variables.   (4.9† vs 5.0† vs 4.4) vs (4.2 vs 4.2)  † indicates statistically different (p<0.05) from Control Groups A and B | | | | | | | | | | | | | | | | | | |  |
| Ouimet 2015  (adults) | Knowledge and skills | Abilities in critical appraisal (14 questions, using practical scenarios and MCQs, measuring the basic knowledge and skills needed before adopting an evidence-informed policy practice).  Score: mean percentage of pre-post improvement, defined as = a-b/c)*100.  a = mean number of wrong answers in pre-test that were converted into correct ones in the post-test; b = mean number of correct answers in pre-test that were converted into wrong ones in post-test; and c = potential for improvement (total number of questions in the test (14) minus the mean number of questions answered correctly in both pre-and post-tests). | Pre- and Post-test | 26 (90%) (n=13, n=13) |  | | | | | | | | **Pre-post improvement**  **Intervention vs comparison** | | | | | | | | **P value** | | |  |
|  |  |  |  |  | A) Mean number of wrong answers converted to correct ones at post-test (SD) | | | | | | | | 4.5 (1.4) vs 2.6 (1.3) | | | | | | | | 0.003 | | |  |
|  |  |  |  |  | B) Mean percentage of pre-post improvement (SD) | | | | | | | | 36.9% (27.5) vs 11.3% (19.1) | | | | | | | | 0.013 | | |  |
|  |  |  |  |  | C) Mean pre-post difference (SD) (maximum of 14) | | | | | | | | +3.6 (2.2) vs +1 (1.8) | | | | | | | | 0.006 | | |  |
| Matic-Strametz 2013  (adults) | Knowledge and skills | Knowledge in Evidence-Based Medicine† (20 single-best / short answer questions). Score: 1 to 3 points (depending on difficulty. Higher score indicates greater skill. | Pre-, During (after 12 hr of seminar) and Post-test | 83 (95%) (n=13, n=70) |  | | | | | | | | **Intervention vs comparison: mean (SD)** | | | | | | | | **P value** | | |  |
|  |  |  |  |  | Pre | | | | | | | | 2.7 (2.5) vs 2.7 (1.7) | | | | | | | | - | | |  |
|  |  |  |  |  | During | | | | | | | | 8.3 (3.2) vs 3.9 (2.4) | | | | | | | | ≤ 0.001 | | |  |
|  |  |  |  |  | Post | | | | | | | | 7.9 (3.8) vs 3.7 (2.3) | | | | | | | | ≤ 0.001 | | |  |
|  |  |  |  |  |  | | | | | | | | **During vs post-test scores: mean (SD)** | | | | | | | | **P value** | | |  |
|  |  |  |  |  | Intervention | | | | | | | | 8.3 (3.2) vs 2.7 (2.5) | | | | | | | | ≤ 0.001 | | |  |
|  |  |  |  |  | Comparison | | | | | | | | 3.9 (2.4) vs 2.7 (1.7) | | | | | | | | not reported | | |  |
|  |  |  |  |  |  | | | | | | | | **Post-test vs pre-test scores: mean (SD)** | | | | | | | | **P value** | | |  |
|  |  |  |  |  | Intervention | | | | | | | | 7.9 (3.8) vs 2.7 (2.5) | | | | | | | | ≤0.001 | | |  |
|  |  |  |  |  | Comparison | | | | | | | | 3.7 (2.3) vs 2.7 (1.7) | | | | | | | | not reported | | |  |
|  | Knowledge and skills | Critical appraisal skills† (35 questions, RCTs appraised with a checklist). Score: 1 to 3 points (depending on difficulty). Higher score indicates greater skill. | Pre-, During (after 12 hr of seminar), and Post-test | 83 (95 %) (n=13, n=70) |  | | | | | | | | **Intervention vs comparison: mean (SD)** | | | | | | | | **P value** | | |  |
|  |  |  |  |  | Pre | | | | | | | | 14.6 (6.3) vs 14.4 (5.6) | | | | | | | | - | | |  |
|  |  |  |  |  | During | | | | | | | | 27.5 (6.4) vs. 15.5 (4.6) | | | | | | | | ≤ 0.01 | | |  |
|  |  |  |  |  | Post | | | | | | | | 24.1 (6.7) vs. 16.2 (5.9) | | | | | | | | ≤ 0.01 | | |  |
|  |  |  |  |  |  | | | | | | | | **During vs post-test scores: mean (SD)** | | | | | | | | **P value** | | |  |
|  |  |  |  |  | Intervention | | | | | | | | 27.5 (6.4) vs 14.6 (6.3) | | | | | | | | ≤ 0.001 | | |  |
|  |  |  |  |  | Comparison | | | | | | | | 15.5 (4.6) vs 14.4 (5.6) | | | | | | | | not significant | | |  |
|  |  |  |  |  |  | | | | | | | | **Post-test vs pre-test scores: mean (SD)** | | | | | | | | **P value** | | |  |
|  |  |  |  |  | Intervention | | | | | | | | 24.1 (6.7) vs 14.6 (6.3) | | | | | | | | <0.01 | | |  |
|  |  |  |  |  | Comparison | | | | | | | | 16.2 (5.9) vs 14.4 (5.6) | | | | | | | | not significant | | |  |
|  | Knowledge and skills | Ability to transfer acquired health literacy information (participants delivered a 1 hr lesson plan to Yrs 8 and 9 students).  Score: evaluated independently by two observers. | Post- test (14 days after intervention) | 12 (14%) (n=12, n=0) | Intervention group only (transfer of acquired information related only to the students who had received the intervention)  Lesson plans (max 20 points): mean (SD) Observer 1: 16.2 (2.1) Observer 2: 15.4 (1.9) Teaching performance (max 50 points): mean (SD) Observer 1: 30.0 (3.7); Observer 2: 29.3 (4.4) | | | | | | | | | | | | | | | | | | |  |
| Steckelberg 2009  (adolescents) | Knowledge and skills | Competence in critical health literacy† (The Critical Health Competence Test, CHC Test, 72 items across 4 scenarios).  Score: no details reported | Post-test | 255 (97%) (n=37, n=218) | Intervention vs comparison  mean (SD), p value 597 (79) vs 483 (94), p < 0.01 | | | | | | | | | | | | | | | | | | |  |
| Berger 2010  (adults) | Knowledge and skills | Assessment of EBM competencies† (19 items on five areas of EBM competencies: question formulation, searching, reading and understanding, calculation, communicating study results.  Score: range from 0 to 19, higher values indicating higher competence. | Post-test (for Intervention; Pre and post collected for comparison) | 166 (81%) (n=123, n=43†)  †pre- and post-tests n=22, post-test only n=21 | Intervention vs comparison  Mean score (SD)  14.7 (3.0) vs 14.4 (3.3)  “Differences between the scores of study participants and students were not statistically significant” (no further details reported) | | | | | | | | | | | | | | | | | | |  |
|  | Skills and behaviour (intervention group only) | Long-term implementation of EBM skills (telephone interviews 6 months post intervention; behaviour categorized into: 1) use of critical appraisal skills; 2) activation of participants to take part in health decision making.  Score: Level 0 (no implementation) - Level 4 (almost complete implementation). | Post-test | 129 (63%) (n=129, n=0) | Level: total n (%)  Level 0: 45 (35)  Level 1: 10 (8)  Level 2: 39 (30)  Level 3: 14 (11)  Level 4: 21 (16)  Additional uptake of advocacy activities: 30 (23%) | | | | | | | | | | | | | | | | | | |  |
| Leshowitz 2002  (adults) | Knowledge and skills | Ability in methodological reasoning (written response to open-ended question)  Score: rated from 0 to maximum 3  Range: 0-2 | Post-test | 177 (78%) (n=59, n=118) | **Intervention vs comparison: mean (SD)** | | | | | | | | | | | | | | | | **P value** | | |  |
|  |  |  |  |  | 1.2 (0.5) vs 0.6 (0.4) | | | | | | | | | | | | | | | | <0.01 | | |  |
|  | Knowledge and skills | Ability to evaluate study quality (written response to open-ended question)  Score: scale ranging from –8 (very poorly done), through 0 (no opinion), to 8 (very well done). | Post-test | 177 (78%) (n=59, n=118) | **Intervention vs comparison: mean (SD)** | | | | | | | | | | | | | | | | **P value** | | |  |
|  |  |  |  |  | –3.9 (3.1) vs 0.1 (1.5) | | | | | | | | | | | | | | | | <0.01 | | |  |
|  | Knowledge and skills | Ability to judge information bias (the degree to which participants rated the article that supported their initial position as being of higher quality than the article contrary to their initial position).  Score: Likert scale ranging from 1 (strongly disagree) to 5 (strongly agree: indicating a lower ability to judge information bias - an increased preference for the article that shared their initial attitude). | Pre- and Post- test | 177 (78%) (n=59, n=118) | **Absolute scale** | | | | | | | | **Mean (SD) compared to zero** | | | | | | | | **P value** | | |  |
|  |  |  |  |  | Intervention | | | | | | | | 0.1 (2.0) vs 0 | | | | | | | | >0.05 | | |  |
|  |  |  |  |  | Comparison | | | | | | | | 0.9 (2.4) vs 0 | | | | | | | | <0.01 | | |  |
|  |  |  |  |  |  | | | | | | | | **Effect size: Intervention vs comparison** | | | | | | | | **P value** | | |  |
|  |  |  |  |  |  | | | | | | | | 0.4 | | | | | | | | < 0.05 | | |  |
| Rowe 2015  (adults) | Knowledge and skills | Abilities in critical thinking† (15 questions, mostly short-answer responses, including ability to interpret graphs and equations, solve basic math problems, identify logical fallacies, recognize when additional information might be needed to evaluate a claim, understand the limitations of correlational data, and develop alternative explanations for a claim).  Score reference:  National average pre-test CAT score: 19.04 | Pre- and Post-test (from 6 semesters; post-test only from 2 semesters) | 475 (100%) (n=272, n=203) | Intervention vs comparison  Post-test only CAT scores: mean ± 1 SE, p value  19.8 (0.4) vs 14.8 (0.4), p < 0.001  Pre- vs post-test CAT scores:  Intervention: 15.5 (0.3) vs 19.8 (0.4), p < 0.001  Comparison: 14.2 (0.6) vs 14.6 (0.7), p = 0.43 | | | | | | | | | | | | | | | | | | |  |
| Leshowitz 1993  (adolescents) | Knowledge and skills | Abilities in critical thinking and scientific reasoning (short-answer test: Part 1 - advertisement from a magazine or newspaper, Part 2 - article in which scientific data were summarised. Two different tests provided pre and post for intervention group, control received post-test only).  Score: judged on subject's overall response to the test items using 3 subtests on 2 point scale: 1. Claim, 2. Graph, 3. Proof. | Post- test (for comparison only; pre- and post- for intervention) | 55 (100%) (n=22, n=33) | Intervention vs comparison: mean  A 2 (group: control vs. pre-test-treatment) X 3 (type of subtest) and a 2 (group: control vs. post-test-treatment) X 3 (type of subtest) analysis of variance to contrast the performance of the control group with the treatment group on pre- and post-tests. The type of subtest was treated as a repeated measure. | | | | | | | | | | | | | | | | | | |  |
|  |  |  |  |  |  | **Range** | | | | **Intervention Pre** | | | **Intervention Post** | | | | **Comparison Post** | | | | **P value** | | |  |
|  |  |  |  |  | 1. Total |  | | | | 1.7 | | | 3.6 | | | | 2.3 | | | | < 0.01 | | |  |
|  |  |  |  |  | 2. Claim | 0 to 3.00 | | | | 1.2 | | | 1.5 | | | | 1.5 | | | | < 0.01 | | |  |
|  |  |  |  |  | 3. Graph | 1.75 to 5.75 | | | | 0.1 | | | 1.2 | | | | 0.1 | | | | < 0.01 | | |  |
|  |  |  |  |  | 4. Proof | 0 to 3.75 | | | | 0.5 | | | 0.9 | | | | 0.7 | | | | < 0.01 | | |  |
|  |  |  |  |  | **Intervention** | | | | | **Intervention vs Comparison (post)** | | | | | | | **Difference** | | | | **P value** | | |  |
|  |  |  |  |  | Pre | | | | | 1.7 vs 2.3 | | | | | | | 0.6 | | | | <0.05 | | |  |
|  |  |  |  |  | Post | | | | | 3.6 vs 2.3 | | | | | | | 1.3 | | | | <0.01 | | |  |
|  |  |  |  |  | **Before vs After (intervention pre vs post)** | | | | | | | | | | | | | | | | | | |  |
|  |  |  |  |  | Total Results | | | | | 1.7 vs 3.6 | | | | | | | +1.9 | | | | <0.01 | | |  |
| Derry 1998  (adolescents) | Knowledge and skills | Ability in statistical reasoning (two tasks, each consisting of a 3-page printed research debate dialogue adapted from a television program scenario, followed by questions about the dialogue.  Score: detailed scoring system, weighting of categories decided prior to analysis, with point values (positive or negative) based on the researchers' consensus view of the category's importance to the scenarios, maximum 13. | Pre- and Post-test | Not reported | Intervention vs comparison (2 experimental groups vs 8 comparison groups) Means 3.6 vs 2.3   Adjusted post-test scores Individual testing: Scenario 1; Scenario 2 5.0 vs 3.2; 2.1 vs 1.5 Group testing: Scenario 1; Scenario 2 4.3 vs 3.1; 3.1 vs 1.4  Adjusted percentage of inappropriate responses  Individual testing: Scenario 1; Scenario 2 18% vs 22%; 39% vs 44%  Group testing: Scenario 1; Scenario 2 6 % vs 40%, 43% vs 64%  Study notes that: “there were no statistically significant results.” | | | | | | | | | | | | | | | | | | |  |

* Assume post-test performed immediately or shortly after intervention, unless otherwise indicated.

† Measure has been validated in some manner.

MCQ: Multiple Choice Question; EBM: Evidence-Based Medicine
