# Supplementary material for: Educational interventions to improve people’s understanding of key concepts in assessing the effects of health interventions: a systematic review
Source: Syst Rev. 2018 May 2;7:68. doi: 10.1186/s13643-018-0719-4 (PMC5930693; doi:10.1186/s13643-018-0719-4)
Supplement: Supplementary file 3 — Table S3. Results for secondary outcomes from included studies (all study designs; randomised trials presented first). (DOCX 58 kb) [file 13643_2018_719_MOESM3_ESM.docx]

**Table 3 Results of secondary outcomes from included studies** (all study designs; randomised trials presented first)

| **Author, Year; (participant age group)** | **Outcome(s) measured** | **Measure/s used** | **Pre- and/or post-test*** | **Numbers analysed** Total n % (intervention n, comparison n) | **Results** | | | | | | | | | | | | | | | | | | | | | | | | | |
| --- | --- | --- | --- | --- | --- | --- | --- | --- | --- | --- | --- | --- | --- | --- | --- | --- | --- | --- | --- | --- | --- | --- | --- | --- | --- | --- | --- | --- | --- | --- |
| **RANDOMISED TRIALS** | | | | | | | | | | | | | | | | | | | | | | | | | | | | | | |
| Nsangi 2017a  (children) | (Intended) Behaviour | 3 questions using a 5 option Likert scale†. | Post-test | 10183 (81%) (n=5753, n=4430) | Intervention vs comparison  n (%), Odds ratio  1. Likely or very likely to ask about the basis of a claim  3731 (64.9%) vs 2440 (55.1%), 1.56 (95% CI 1.29 to 1.88)  Adjusted difference in proportions: 10.6% (95% CI 6.2% to 14.7%)  2. Likely or very likely to find out if a claim is based on appropriate research  3114 (54.1%) vs 1967 (44.4%), 1.54 (95% CI 1.29 to 1.84)  Adjusted difference in proportions: 10.8% (95% CI 6.3% to 15.1%)  3: Likely or very likely to participate in appropriately performed research  3201 (55.6%) vs 2163 (48.8%), 1.37 (95% CI 1.16 to 1.62)  Adjusted difference in proportions: 7.8% (95% CI 3.7% to 11.9%) | | | | | | | | | | | | | | | | | | | | | | | | | |
|  | Confidence | 4 questions using a 5 option Likert scale†. | Post-test | 10183 (81%) (n=5753, n=4430) | Intervention vs comparison  n (%), Odds ratio  1. Very easy or easy (to assess if a claim is based on appropriate research)  3244 (56.4%) vs 1886 (42.6%), 1.83 (95% CI 1.55 to 2.16)  Adjusted difference in proportions: 15.0% (95% CI 10.9% to 19.0%)  2. Very easy or easy (to find information based on appropriate research)  2238 (50.5%) vs 3069 (53.3%),1.13 (95% CI 0.96 to 1.33)  Adjusted difference in proportions: 3.0% (95% CI -1.0% to 7.0%)  3. Very easy or easy (to assess how reliable are the results of research)  2112 (36.7%) vs 1777 (40.1%), 0.84 (95% CI 0.73 to 0.96)  Adjusted difference in proportions: -4.1% (95% CI -1.0% to -7.3%)  4. Very easy or easy (to determine if the research results are relevant to me)  2727 (47.4%) vs 2002 (45.2%), 1.08 (95% CI 0.93 to 1.25)  Adjusted difference in proportions: 1.9% (95% CI -1.8% to 5.6%) | | | | | | | | | | | | | | | | | | | | | | | | | |
|  | Attitude | 4 questions.  Score: 5 option Likert scale†. | Post-test | 10183 (81%) (n=5753, n=4430) | Intervention vs comparison  n (%), Odds ratio  1. Like or like a lot (school)  5402 (93.9%) vs 4171 (94.2%), 0.96 (95% CI 0.69 to 1.34)  Adjusted difference in proportions: -0.2% (95% CI -2.4% to 1.4%)  2. Like or like a lot (science as a subject)  5285 (91.9%) vs 4087 (92.3%), 1.08 (95% CI 0.78 to 1.33)  Adjusted difference in proportions: -0.1% (95% CI -2.7% to 1.8%)  3. Very helpful or helpful (to what is being learned in school)  5265 (91.5%) vs 4043 (91.3%), 0.98 (95% CI 0.72 to 1.33)  Adjusted difference in proportions: 0.6% (95% CI -2.2% to 2.0%)  4. Very helpful or helpful (to what is being learned in science)  5169 (89.8%) vs 4207 (95.0%); 0.96 (95% CI 0.70 to 1.32)  Adjusted difference in proportions: 0.2% (95% CI - 2% to 1.2%) | | | | | | | | | | | | | | | | | | | | | | | | | |
|  | Satisfaction | 4 questions using a 4 option Likert scale†. | Post-test | 5753 (46%)  (n=5753, n=0) | Intervention vs comparison  n (%)  a. Like or like a lot (learning from the intervention): 5394 (93.8%)  b. Very helpful or helpful (learning from the intervention): 5148 (89.5%)  c. Very easy or easy (learning from the intervention): 4864 (84.5%)  d. Trust it very much or trust it (learning from the intervention): 5039 (87.6%) | | | | | | | | | | | | | | | | | | | | | | | | | |
| Semakula 2017  (children) | (Intended) Behaviour | 3 questions.  Score: 5 option Likert scale†. | Post-test | 561 (83%) (n=288, n=273) | Intervention vs comparison  n (%), Odds ratio  1. Likely or very likely to ask about the basis of a claim  219 (75.0%) vs 210 (76.9%), 0.93 (95% CI 0.63 to 1.38) p=0.72  Adjusted difference in proportions: -1.3% (95% CI -7.9% to 6.2%)  2. Likely or very likely to find out if a claim is based on appropriate research  217 (75.3%) vs 194 (71.1%), 1.23 (95% CI 0.85 to 1.80) p=0.27  Adjusted difference in proportions: 4.1% (95% CI -3.1% to 11.6%)  3: Likely or very likely to participate in appropriately performed research  238 (82.6%) vs 224 (82.1%), 1.03 (95% CI 0.66 to 1.59) p=0.90  Adjusted difference in proportions: 0.4% (95% CI -5.7% to 6.9%) | | | | | | | | | | | | | | | | | | | | | | | | | |
|  | Confidence | 4 questions using a 5 option Likert scale†. | Post-test | 561 (83%) (n=288, n=273) | Intervention vs comparison  n (%), Odds ratio, p value  1. Very easy or easy (to assess if a claim is based on appropriate research)  185 (64.2%) vs 133 (48.7%), 1.92 (95% CI 1.36 to 2.70) p=0.00018  Adjusted difference in proportions: 15.5% (95% CI 7.4% to 23.6%)  2. Very easy or easy (to find information based on appropriate research)  208 (72.2%) vs 155 (56.8%), 1.98 (95% CI 1.39 to 2.81) p= 0.00015  Adjusted difference in proportions: 15.4% (95% CI 7.6% to 23.3%)  3. Very easy or easy (to assess how reliable are the results of research)  205 (71.2%) vs 114 (41.8%), 3.42 (95% CI 2.41 to 4.86) p<0.00001  Adjusted difference in proportions: 29.4% (95% CI 21.6% to 37.3%)  4. Very easy or easy (to determine if the research results are relevant to me)  222 (77.1%) vs 182 (66.7%), 1.66 (95% CI 1.14 to 2.41) p=0.00812  Adjusted difference in proportions: 10.2% (95% CI 3.0% to 17.8%) | | | | | | | | | | | | | | | | | | | | | | | | | |
|  | Satisfaction | 4 questions using 4 option Likert scale†. | Post-test | 288 (43%) (n=288, n=0) | Intervention vs comparison  n (%)  a. Like or like a lot (learning from the intervention): 274 (95.1%)  b. Very helpful or helpful (learning from the intervention): 276 (95.8%)  c. Very easy or easy (learning from the intervention): 220 (76.4%)  d. Trust it very much or trust it (learning from the intervention): 263 (91.3%) | | | | | | | | | | | | | | | | | | | | | | | | | |
| Kruse 2000  (adults) | Attitude | Attitude toward research and randomised trials† (32 statements in Likert-type format). Score: 0 to 4-point scale, max 128 points (more positive attitude). | Pre- and Post-test (2 weeks post intervention) | 313 (73%) (n=75, n=78, n=83, n=77)  Total (groups A, B, C, Control.) | Intervention vs comparison | | | | | | | | | | | | | | | | | | | | | | | | | |
|  |  |  |  |  |  | | **Mean change pre to post (SD)** | | | | | | | | | | **Difference** | | | | | | | **P value** | | | | | | |
|  |  |  |  |  | A. Leaflet | | 71.1 (15.0) vs 71.5 (15.6) | | | | | | | | | | 0.4 | | | | | | | 0.93 | | | | | | |
|  |  |  |  |  | B. Brochure | | 73.3 (16.4) vs 74.4 (20.8) | | | | | | | | | | 1.1 | | | | | | | 0.80 | | | | | | |
|  |  |  |  |  | C. Booklet | | 70.4 (13.4) vs 75.1 (15.5) | | | | | | | | | | 4.7 | | | | | | | 0.01 | | | | | | |
|  |  |  |  |  | Control | | 71.1 (14.1) vs 71.7 (13.9 | | | | | | | | | | 0.6 | | | | | | | reference | | | | | | |
| Santesso 2015  (adults) | Satisfaction | Accessibility and usability of findings† (6 items; 5 items on a 7-point Likert scale from 1 - strongly disagree, to 7 - strongly agree  Score: % of participants who selected each result option for each question. | Post-test | 143 (74 %) (n=74, n=69) | Intervention vs comparison  “All comparisons were statistically significant except for the ease of understanding” | | | | | | | | | | | | | | | | | | | | | | | | | |
|  |  |  |  |  | *Class period* | | | | | | | | | | | | | | | | | | | | | | | | | |
|  |  |  |  |  | **1** | **2** | | | | **3** | | | | **4** | | | | | **5** | | | | | **6** | | | | **7** | | |
|  |  |  |  |  | 1. Information easy to find | | | | | | | | | | | | | | | | | | | | | | | | | |
|  |  |  |  |  | 4 vs 3 | 5 vs 14 | | | | 5 vs 10 | | | | 5 vs 9 | | | | | 21 vs 22 | | | | | 49 vs 35 | | | | 8 vs 7 | | |
|  |  |  |  |  | 2. Summary presents important effects | | | | | | | | | | | | | | | | | | | | | | | | | |
|  |  |  |  |  | 5 vs 2 | 5 vs 15 | | | | 4 vs 10 | | | | 4 vs 8 | | | | | 19 vs 18 | | | | | 49 vs 38 | | | | 14 vs 11 | | |
|  |  |  |  |  | 3. Information is reliable | | | | | | | | | | | | | | | | | | | | | | | | | |
|  |  |  |  |  | 5 vs 2 | 0 vs 2 | | | | 10 vs 5 | | | | 6 vs 12 | | | | | 28 vs 23 | | | | | 39 vs 42 | | | | 11 vs 12 | | |
|  |  |  |  |  | 4. Information is easy to understand | | | | | | | | | | | | | | | | | | | | | | | | | |
|  |  |  |  |  | 4 vs 2 | 8 vs 7 | | | | 8 vs 11 | | | | 1 vs 0 | | | | | 25 vs 18 | | | | | 47 vs 55 | | | | 7 vs 6 | | |
|  |  |  |  |  | 5. Presentation to help make a decision | | | | | | | | | | | | | | | | | | | | | | | | | |
|  |  |  |  |  | 5 vs 4 | 14 vs 7 | | | | 15 vs 8 | | | | 8 vs 7 | | | | | 21 vs 24 | | | | | 37 vs 34 | | | | 8 vs 7 | | |
| Woloshin 2007a  (adults) | Confidence | Interest and confidence in interpreting medical statistics† (during eligibility interview and post-educational intervention survey). Score: 0 to 100 (higher interest or confidence). | Pre- and Post-test | 322 (96%) (n=153, n=169) |  | | | | | | | **Pre** | | | **Post** | | | | | **Change** | | | | | **Difference** | | | | **P value** | |
|  |  |  |  |  | 1. Interest in interpreting medical statistics (mean scores) | | | | | | | | | | | | | | | | | | | | | | | | | |
|  |  |  |  |  | Intervention | | | | | | | 57 | | | 61 | | | | | +4 | | | | | 6 | | | | 0.004 | |
|  |  |  |  |  | Comparison | | | | | | | 58 | | | 56 | | | | | -2 | | | | |  |  |  |  |  |  |
|  |  |  |  |  | 2. Confidence in interpreting medical statistics (mean scores) | | | | | | | | | | | | | | | | | | | | | | | | | |
|  |  |  |  |  | Intervention | | | | | | | 65 | | | 67 | | | | | +2 | | | | | 2 | | | | 0.36 | |
|  |  |  |  |  | Comparison | | | | | | | 65 | | | 69 | | | | | +4 | | | | |  |  |  |  |  |  |
|  | Satisfaction | Satisfaction with intervention: participants rated each booklet on various dimensions, using multiple choice options. Score: response n | Post-test | 322 (96%) (n=153, n=169) | Intervention vs comparison  Booklet Use Questions | | | | | | | | | | | | | | | | | | | | | | | | | |
|  |  |  |  |  |  | | | | **≤10 min** | | | | **30 min** | | | | | | | | **≥1 h** | | | | | | | | | |
|  |  |  |  |  | 1. Time | | | | 1 vs 7 | | | | 27 vs 65 | | | | | | | | 72 vs 28, p<0.001 | | | | | | | | | |
|  |  |  |  |  |  | | | | **Very easy** | | | | **Easy** | | | | | | | | **Hard** | | | | | **Very hard** | | | | |
|  |  |  |  |  | 2. Difficulty | | | | 55 vs 19 | | | | 55 vs 19 | | | | | | | | 3 vs 0 | | | | | 0 vs 0 | | | | |
|  |  |  |  |  |  | | | | **Very helpful** | | | | **Helpful** | | | | | | | | **A little** | | | | | **Not at all** | | | | |
|  |  |  |  |  | 3. Helpfulness | | | | 51 vs 16 | | | | 40 vs 53 | | | | | | | | 9 vs 30 | | | | | 0 vs 1 | | | | |
|  |  |  |  |  |  | | | | 91% found the primer “helpful” or “very helpful” versus 69% for the control booklet (p< 0.001).  “All ratings were significantly higher for the primer group than for the control group.” | | | | | | | | | | | | | | | | | | | | | |
|  |  |  |  |  |  | | | | **Definitely yes** | | | | **Probably yes** | | | | | | | | **Probably no** | | | | | **Definitely no** | | | | |
|  |  |  |  |  | Refer to later | | | | 28 vs 10 | | | | 52 vs 41 | | | | | | | | 14 vs 44 | | | | | 5 vs 5 | | | | |
|  |  |  |  |  | Recommend to others | | | | 45 vs 26 | | | | 42 vs 43 | | | | | | | | 11 vs 26 | | | | | 3 vs 5 | | | | |
|  |  |  |  |  | Read before | | | | 22 vs 8 | | | | 49 vs 34 | | | | | | | | 23 vs 49 | | | | | 6 vs 9 | | | | |
|  |  |  |  |  | Learned | | | | 54 vs 28 | | | | 37 vs 38 | | | | | | | | 8 vs 26 | | | | | 1 vs 7 | | | | |
| Woloshin 2007b  (adults) | Confidence | Interest and confidence in interpreting medical statistics† (during eligibility interview and post-educational intervention survey). Score: 0 to 100 (higher interest or confidence). | Pre- and Post-test | 200 (91%) (n=98, n=102) |  | | | | **Pre** | | | **Post** | | | **Change** | | | | | | **Difference** | | | | | **P value** | | | | |
|  |  |  |  |  | 1. Interest in interpreting medical statistics (mean scores) | | | | | | | | | | | | | | | | | | | | | | | | | |
|  |  |  |  |  | Intervention | | | | 59 | | | 65 | | | +6 | | | | | | 8 | | | | | 0.004 | | | | |
|  |  |  |  |  | Comparison | | | | 59 | | | 57 | | | -2 | | | | | |  |  |  |  |  |  |  |  |  |  |
|  |  |  |  |  | 2. Confidence in interpreting medical statistics (mean scores) | | | | | | | | | | | | | | | | | | | | | | | | | |
|  |  |  |  |  | Intervention | | | | 62 | | | 64 | | | +2 | | | | | | 4 | | | | | 0.166 | | | | |
|  |  |  |  |  | Comparison | | | | 65 | | | 71 | | | +6 | | | | | |  |  |  |  |  |  |  |  |  |  |
|  | Satisfaction | Satisfaction with intervention (participants asked to rate each booklet on various dimensions, using multiple choice options).  Score: response n | Post-test | 200 (91%) (n=98, n=102) | Intervention vs comparison  Booklet Use Questions | | | | | | | | | | | | | | | | | | | | | | | | | |
|  |  |  |  |  |  | | | | **≤10 min** | | | | **30 min** | | | | | | | | **≥1 h** | | | | | | | | | |
|  |  |  |  |  | 1. Time | | | | 2 vs 4 | | | | 24 vs 48 | | | | | | | | 74 vs 47, p=0.001 | | | | | | | | | |
|  |  |  |  |  |  | | | | **Very easy** | | | | **Easy** | | | | | | | | **Hard** | | | | | | **Very hard** | | | |
|  |  |  |  |  | 2. Difficulty | | | | 53 vs 39 | | | | 53 vs 39 | | | | | | | | 9 vs 1 | | | | | | 1 vs 0 | | | |
|  |  |  |  |  |  | | | | **Very helpful** | | | | **Helpful** | | | | | | | | **A little** | | | | | | **Not at all** | | | |
|  |  |  |  |  | 3. Helpfulness | | | | 37 vs 41 | | | | 57 vs 49 | | | | | | | | 5 vs 11 | | | | | | 0 vs 0 | | | |
|  |  |  |  |  |  | | | | 94% and 90% of participants rated the primer and control booklet “helpful” or “very helpful,” respectively (p= 0.26).  “…the results were not statistically different from the control booklet.” | | | | | | | | | | | | | | | | | | | | | |
|  |  |  |  |  |  | | | | **Definitely yes** | | | | **Probably yes** | | | | | | | | **Probably no** | | | | | | **Definitely no** | | | |
|  |  |  |  |  | Refer to later | | | | 27 vs 25 | | | | 47 vs 57 | | | | | | | | 21 vs 17 | | | | | | 5 vs 1 | | | |
|  |  |  |  |  | Recommend to others | | | | 38 vs 50 | | | | 52 vs 40 | | | | | | | | 6 vs 9 | | | | | | 4 vs 2 | | | |
|  |  |  |  |  | Read before | | | | 34 vs 24 | | | | 41 vs 52 | | | | | | | | 22 vs 22 | | | | | | 3 vs 2 | | | |
|  |  |  |  |  | Learned | | | | 68 vs 51 | | | | 24 vs 39 | | | | | | | | 5 vs 8 | | | | | | 3 vs 2 | | | |
| Austvoll-Dahlgren 2012  (adults) | Attitude | Evaluating beliefs about searching for health information. Three variables were rated using direct and indirect measures: attitude, subjective norms, and perceived behavioural control.  Score: rating was measured against ratings of the same material made by experts. (higher values indicate more favourable attitudes, greater social pressure and higher perceived behavioural control). | Post-test | 67 (70%) (n=28, n=39) | Intervention vs comparison | | | | | | | | | | | | | | | | | | | | | | | | | |
|  |  |  |  |  |  | | | | | | **Mean (SD)** | | | | | | | | | | | | **Difference** | | | **95% CI** | | | | **P value** |
|  |  |  |  |  | Direct measures (score range: 1-7) | | | | | | | | | | | | | | | | | | | | | | | | | |
|  |  |  |  |  | 1. Intention | | | | | | 6.1 (1.1) vs 5.8 (1.1) | | | | | | | | | | | | 0.3 | | | -0.2 to 0.9 | | | | 0.20 |
|  |  |  |  |  | 2. Direct attitude | | | | | | 5.8 (1.1) vs 5.2 (1.2) | | | | | | | | | | | | 0.6 | | | 0.1 to 1.2 | | | | 0.03 |
|  |  |  |  |  | 3. Direct subjective norm | | | | | | 3.4 (1.3) vs 3.6 (1.2) | | | | | | | | | | | | -0.2 | | | -0.8 to 0.4 | | | | 0.49 |
|  |  |  |  |  | 4. Direct perceived behavioural | | | | | | 5.6 (1.1) vs 5.3 (1.1) | | | | | | | | | | | | 0.4 | | | -0.2 to 1.0 | | | | 0.15 |
|  |  |  |  |  | Overall composite indirect measures  (score range A: -84 to +84, B and C: -63 to +63) | | | | | | | | | | | | | | | | | | | | | | | | | |
|  |  |  |  |  | 5. Overall indirect attitude | | | | | | 53.7 (24.2) vs 50.8 (24.8) | | | | | | | | | | | | 2.9 | | | -9.3 to 15 | | | | 0.64 |
|  |  |  |  |  | 6. Overall indirect subjective norm | | | | | | (14.5) vs 10.3 (12.5) | | | | | | | | | | | | 3.6 | | | -3.0 to 10.3 | | | | 0.28 |
|  |  |  |  |  | 7. Overall indirect perceived behavioural control | | | | | | -4.4 (13.4) vs -3.1 (17.3) | | | | | | | | | | | | -1.3 | | | -9.1 to 6.6 | | | | 0.74 |
|  | Confidence | Overall activation and participants’ self-management abilities† (13 items, in 4 domains: 1. Believing the patient role is important, 2. Having confidence and knowledge necessary to taking action, 3. Actually taking action to improve one’s health, 4. Staying the course under stress). Score: 0 to 100 (high activation) | Post-test | 67 (70%) (n=28, n=39) | Intervention vs comparison Test scores, difference, p value 66.5 vs 61.9, 4.6, p= 0.20 | | | | | | | | | | | | | | | | | | | | | | | | | |
|  | Satisfaction | Satisfaction with web-portal† (4 questions to assess website usefulness, usability and credibility). Score: 1 to 7, higher values indicating greater satisfaction. | Post-test | 28 (29%) (n=28, n=0) | Intervention group only Mean score (SD) Mean usefulness: 4.7 (SD 1.1) Mean usability: 4.1 (SD 1.0)  Mean credibility 4.8 (SD 0.9)  “Satisfaction with the web portal was good.” | | | | | | | | | | | | | | | | | | | | | | | | | |
| Tait 2015  (children and adults) | Satisfaction | Preference of format (participants shown other format at end of study and asked which they preferred). | Post-test | Not reported | Intervention format vs comparison format Children: 67.9% vs 32.1% Parents: 62.4% vs 37.6% | | | | | | | | | | | | | | | | | | | | | | | | | |
|  | Satisfaction | Perception of information delivery, satisfaction and information preference † (survey using numbers scales and Likert scales).  Score: 0-10 ("extremely satisfied"), and the number of people indicating a positive Likert response to intervention. | Post-test | 283 (100%) (n=140, n=143)  Parents: 148 (n=73, n=75) Children: 135 (n=67, n=68) | Intervention vs comparison | | | | | | | | | | | | | | | | | | | | | | | | | |
|  |  |  |  |  |  | | | | | **Children** | | | | | | **P value** | | | | | | **Parents** | | | | | | **P value** | | |
|  |  |  |  |  | 1. Information quality: mean (SD) | | | | | 8.7(1.4) vs 8.2 (1.6) | | | | | | not reported | | | | | | 9.4 (1.0) vs 9.0 (1.2) | | | | | | <0.05 | | |
|  |  |  |  |  | 2. Ability to follow information: mean (SD) | | | | | 8.2 (1.8) vs 7.2 (1.8) | | | | | | <0.05 | | | | | | 9.3 (1.1) vs 8.8 (1.6) | | | | | | <0.05 | | |
|  |  |  |  |  | 3. Effectiveness of presentation: ‘Extremely effective’, n (%) | | | | | 45 (68.2) vs 29 (42.0) | | | | | | <0.05 | | | | | | 67 (91.8) vs 56 (74.7) | | | | | | <0.05 | | |
|  |  |  |  |  | 4. Amount of information: ‘Just right’, n (%) | | | | | 65 (98.5) vs 60 (87.0) | | | | | | <0.05 | | | | | | 68 (93.2) vs 69 (92.0) | | | | | | not reported | | |
|  |  |  |  |  | 5. Clarity of information: ‘Very clear’, n (%): | | | | | 48 (72.7) vs 28 (40.6) | | | | | | 0.05 | | | | | | 62 (82.7) vs 62 (84.9) | | | | | | not reported | | |
|  |  |  |  |  | Overall satisfaction with iPad graphics and interactivity: mean (SD) | | | | | Range: 8.6 (1.5) to 9.6 (0.7) | | | | | | | | | | | | | | | | | | | | |
| Welch 2014  (adults) | Satisfaction | Perception of intervention web-based modules (2 questions with 3 options - extremely/mostly/somewhat; 2 questions with 2 options - agree/disagree). Score: response n (%) | Post-test | 82 (17%) (n=82, n=0) | Intervention group only: n (%) | | | | | | | | | | | | | | | | | | | | | | | | | |
|  |  |  |  |  |  | | | **Extremely easy / helpful** | | | | **Mostly easy / helpful** | | | | | | **Somewhat easy / helpful** | | | | | | | **Extremely difficult / unhelpful** | | | | | |
|  |  |  |  |  | Ease of understanding | | | 9 (11.0) | | | | 57 (69.5) | | | | | | 13 (15.9) | | | | | | | 3 (3.7) | | | | | |
|  |  |  |  |  | Helpfulness of information | | | 29 (35.3) | | | | 35 (42.7) | | | | | | 18 (22.0) | | | | | | | 0 (0) | | | | | |
|  |  |  |  |  |  | | | | | | | **Agree** | | | | | | | | | | | | | **Disagree** | | | | | |
|  |  |  |  |  | Intention to recommend | | | | | | | 74 (90.2) | | | | | | | | | | | | | 8 (9.8) | | | | | |
|  |  |  |  |  | Interest in further education | | | | | | | 72 (87.8) | | | | | | | | | | | | | 10 (12.2) | | | | | |
| Barnett 2005  (children) | Satisfaction | Intervention usability determining (with use of questions) whether the children  1. found the forms easy to read 2. if they would help the doctor (by participating in a trial). Score: 1. no details reported 2. total number who would agree to help/participate in a trial | Post-test | 342 (91%) (n=115, n=110, n=117) (assumed, not reported) | A (Q and A format) vs B (Story format) vs C (Text format) 1. Easy to read? No significant difference of the reported ease of reading between the formats (no further details reported). 2. Would students help doctor?  Total number n (%): A vs B vs C 91 (72%) vs 72 (58%) vs 87 (71%) | | | | | | | | | | | | | | | | | | | | | | | | | |
| **OTHER STUDY DESIGNS** | | | | | | | | | | | | | | | | | | | | | | | | | | | | | | |
| Kaelin 2007  (adolescents) | Attitude | Attitudes towards science inquiry, 33 items from a 62-item test.  Score: 33 items, using a six-point Likert scale† ("Strongly disagree" to "Strongly agree"). No further details reported. | Pre- and Post-test | 687 (47%) | Intervention vs comparison  (Exp. Gr. 1 vs Exp. Gr. 2 vs Exp. Gr. 3) vs (Cont. Gr. 1 vs Cont. Gr. 2)  (150.5†, 146.9, 141.4§) vs (142.6, 142.9)  † indicates statistically different (p<0.05) from Control Groups A and B § indicates statistically different (p<0.05) from Experimental Group 2 | | | | | | | | | | | | | | | | | | | | | | | | | |
|  | Attitude | Attitudes towards science inventory, 7 items from a 62-item test† (perceived abilities in science generally, interest in science generally, and understanding of epidemiology specifically). Score: 7 items (using a six-point Likert scale from "Strongly disagree" to "Strongly agree"). No further details reported. | Pre- and Post-test | 962 (66%) | Intervention vs comparison  (Exp. Gr. 1 vs Exp. Gr. 2 vs Exp. Gr. 3) vs (Cont. Gr. 1 vs Cont. Gr. 2)  (28.1† vs 27.2 vs 27.1) vs (26.5 vs 26.7) † indicates statistically different (p<0.05) from Control Groups A and B | | | | | | | | | | | | | | | | | | | | | | | | | |
|  | Perception of knowledge and/or abilities | Students' perceived understanding of “enduring understandings” † (5 items from a 62 item test) Score: 5 items (using a five point Likert scale of "Definitely do not understand" to "Definitely understand"). No further details reported. | Pre- and Post-test | 929 (63%) | Intervention vs comparison  (Exp. Gr. 1 vs Exp. Gr. 2 vs Exp. Gr. 3) vs (Cont. Gr. 1 vs Cont. Gr. 2)  (21.1†, 18.7‡, 18.1‡) vs (17.9, 17.5)  † indicates statistically different (p<0.05) from Control Groups A and B ‡ indicates statistically different (p<0.05) from Control Group B | | | | | | | | | | | | | | | | | | | | | | | | | |
|  | Perception of knowledge and/or abilities | Participants perceived abilities in indicators of scientific literacy†, 6 items from a 62 item test to address several end-points that could reasonably be expected to improve with exposure to epidemiology teaching. Score: 6 items, using a five-point Likert scale from "Definitely No" to "Definitely Yes". No further details reported. | Pre- and Post-test | 948 (65%) | Intervention vs comparison  (Exp. Gr. 1 vs Exp. Gr. 2 vs Exp. Gr. 3) vs (Cont. Gr. 1 vs Cont. Gr. 2) (24.1†, 23.3‡, 23.1‡) vs (22.8, 22.4)  † indicates statistically different (p<0.05) from Control Groups A and B ‡ indicates statistically different (p<0.05) from Control Group B | | | | | | | | | | | | | | | | | | | | | | | | | |
| Matic-Strametz 2013  (adults) | Satisfaction | Satisfaction with intervention course  Score: a. number of participants who intend to use similar materials in future teaching  b. participants provided a rating of 1 to 5. | Pre-, During- (12 hours of course), and Post-test (immediately post intervention and 14 days post intervention) | 12 (14%) (n=12, n=0) | Intervention group only a. Number/total group size (%) Post intervention: 10/13 (77%) Post intervention +14 days: 10/12 (83%)  b. Satisfaction (range 1-5) median grade of 2 [good]. No further details provided. | | | | | | | | | | | | | | | | | | | | | | | | | |
|  | Satisfaction | Student satisfaction with PBL sessions (high school students receiving PBL rated the sessions provided by the study participants). Score: range of answers with maximum score "very interesting". No further details provided. | Post-test (14 days after intervention) | 95 (96%)  [students from PBL sessions, NOT from study] | Data from outside study participants: n % Found PBL session interesting: 85 (89%) [Very interesting n=49, Mostly of interest: n=36] Would like to see the lesson in other subjects: 84 (88% of respondents) | | | | | | | | | | | | | | | | | | | | | | | | | |
| Steckelberg 2009  (adolecents) | Satisfaction | Participants' responses to the question “What is your personal benefit of this week?”, referring to the intervention. Score: responses were collected. | Post-test | 21 (8%) (n=21, n=0) | Responses to “What is your personal benefit of this week?”: *1. The difference between relative and absolute risk reduction. 2. Relative and absolute risk reduction. 3. Clarification of misleading framing of results in mass media. 4. I learned a lot and now I can ask my doctor questions more specifically to get better answers. That might help to decide on medications. 5. New knowledge on risks and research methods in medicine. 6. Reasoning about statistics, questions to ask my doctor and knowledge on medical terms. 7. I picked up a lot of new things, which do prepare for visits to my doctor as well as to understand medical research papers. Thanks a lot. 8. A critical view on medical information; the specific reckoning of statistics. 9. Next time I go to the doctors I will listen carefully and ask questions if I do not understand. 10. Medpilot.de; the workbook 11. Critical view on statistics; I’ll take the workbook with me. 12. I will ask my doctor more questions and I will look for more information on prescriptions of medicine, before I take it or do not take it. Now after this course, I know where to get this information. 13. I will read package inserts of drugs more carefully, if I want to take anything. I learned a lot during this week. 14. Better understanding of medical issues, especially research and studies. 15. I know better how to interpret studies and I can mistrust percentages. I found it interesting to get to know how studies are conducted. Now I can better understand package inserts of drugs regarding side effects. 16. I learned a lot this week. I liked the different materials we got. The workbook is very well designed, so I can refer to it at home. 17. I hate to work with computers. 18. Nothing. 19. Nothing.* | | | | | | | | | | | | | | | | | | | | | | | | | |
| Ciarocco 2013  (adults) | Perception of knowledge and/or abilities | Perceptions of participants' own skills in scientific writing (assessed using positively and negatively keyed statements). Score: 1 (strongly disagree) to 7 (strongly agree). No further details reported. | Post-test | 70 (84%) (n=37, n=33) |  | | | | | | **Intervention vs comparison: mean (SD)** | | | | | | | | | | **Difference** | | | | | | **P value** | | | |
|  |  |  |  |  | 1. Efficacy in writing style: | | | | | | 6.5 (0.6) vs 6.1 (0.7) | | | | | | | | | | 0.36 | | | | | | 0.024 | | | |
|  |  |  |  |  | 2. Efficacy writing method sections: | | | | | | 6.5 (0.8) vs 6.1 (0.9) | | | | | | | | | | 0.45 | | | | | | 0.03 | | | |
|  |  |  |  |  | 3. Efficacy for writing results sections: | | | | | | 6.4 (0.8) vs 5.6 (1.3) | | | | | | | | | | 0.77 | | | | | | 0.003 | | | |
|  | Perception of knowledge and/or abilities | Perceptions of skills in research and statistics (assessed using positively and negatively keyed statements).  Score: 1 (strongly disagree) to 7 (strongly agree). No further details reported. | Post-test | 70 (84%) (n=37, n=33) |  | | | | | | **Intervention vs comparison: mean (SD)** | | | | | | | | | | **Difference** | | | | | | **P value** | | | |
|  |  |  |  |  | 1. Research attitudes: | | | | | | 4.0 (1.4) vs 3.6 (1.4) | | | | | | | | | | 0.4 | | | | | | 0.211 | | | |
|  |  |  |  |  | 2. Research skills/abilities: | | | | | | 5.0 (0.9) vs 4.9 (0.8) | | | | | | | | | | 0.1 | | | | | | 0.709 | | | |
|  |  |  |  |  | 3. Research perceived utility: | | | | | | 5.6 (1.0) vs 4.8 (1.1) | | | | | | | | | | 0.8 | | | | | | 0.002 | | | |
|  |  |  |  |  | 4. Statistics attitudes: | | | | | | 5.1 (2.6) vs 3.9 (1.6) | | | | | | | | | | 1.2 | | | | | | 0.020 | | | |
|  |  |  |  |  | 5. Statistics skills/abilities: | | | | | | 5.4 (1.1) vs 4.9 (1.0) | | | | | | | | | | 0.5 | | | | | | 0.028 | | | |
|  |  |  |  |  | 6. Statistics perceived utility: | | | | | | 5.4 (1.0) vs 4.7 (1.2) | | | | | | | | | | 0.7 | | | | | | 0.008 | | | |
| Berger 2010  (adults) | Satisfaction | Acceptability of the intervention (participants evaluated every module of the main course related to their personal learning goals using visual analogue scales). Score: 0 to 100% | Post-test | 129 (63%) (n=129, n=0) | Intervention group only | | | | | | | | | | | | | | | | | | | | | | | | | |
|  |  |  |  |  | **Satisfaction in terms of different learning goals:** | | | | | | | | | | | | | | | | | | | | | | **Mean % (SD)** | | | |
|  |  |  |  |  | Skills in research, critical appraisal and communication, advanced education or understanding of EBM | | | | | | | | | | | | | | | | | | | | | | 80% (4) | | | |
|  |  |  |  |  | Networking, empowerment, implementation, others. | | | | | | | | | | | | | | | | | | | | | | 65% (2) | | | |
| Leshowitz 2002  (adults) | Attitude | Assessment of change in attitude (participants rated how their opinion had changed)  Score: the average of 4 measurements ranging from –8 (more opposed), through 0 (no change), to 8 (more in favour). No further details reported. | Pre- and Post- test | 177 (78%) (n=59, n=118) | **Absolute scale** | | | | | | **Mean (SD) compared to zero** | | | | | | | | | | | | | | | | **P value** | | | |
|  |  |  |  |  | Intervention | | | | | | 0.6 (1.5) vs 0 | | | | | | | | | | | | | | | | < 0.01 | | | |
|  |  |  |  |  | Comparison | | | | | | 1.5 (2.0) vs 0 | | | | | | | | | | | | | | | | < 0.001 | | | |
|  |  |  |  |  |  | | | | | | **Effect size: Intervention vs comparison** | | | | | | | | | | | | | | | | **P value** | | | |
|  |  |  |  |  |  | | | | | | 0.5 | | | | | | | | | | | | | | | | <0.01 | | | |
| Rowe 2015  (adults) | Attitude | Changes in attitude about science in general and controversial scientific theories (20 question Likert-scale survey). Score: no details reported. | Pre- and Post- test | Pre-test  1443 (100%) (n=882, n=561) Post-test  1250 (87%) (n=833, n=417) | Pre and post-test comparison Pooled analysis across all semesters of pre- and post-tests: mean (SD), p value Intervention: 66.2 (0.5) vs 75.5 (0.5), p < 0.001 Comparison: 65.3 (0.6) vs 64.9 (0.7), p = 0.69 | | | | | | | | | | | | | | | | | | | | | | | | | |

* Assume post-test performed immediately or shortly after intervention, unless otherwise indicated.

† Measure has been validated in some manner.

Q and A: Question and Answer
